# Supplementary material for: Altering the Chain Length Specificity of a Lipase from Pleurotus citrinopileatus for the Application in Cheese Making
Source: Foods. 2022 Aug 28;11(17):2608. doi: 10.3390/foods11172608 (PMC9455245; doi:10.3390/foods11172608)
Supplement: Supplementary file 1 [file foods-11-02608-s001.zip › foods-1802791-supplementary.pdf]

foods

Article

Altering the Chain Length Specificity of a Lipase from *Pleurotus citrinopileatus* for the Application in Cheese Making

Niklas Broel [1], Miriam A. Sowa [1], Julia Manhard [2], Alexander Siegl [2], Edgar Weichhard [2], Holger Zorn [1], Binglin Li [3,*] and Martin Gand [1,*]

[1] Institute of Food Chemistry and Food Biotechnology, Justus Liebig University Giessen, 35392 Giessen, Germany
[2] Optiferm GmbH, 87466 Oy-Mittelberg, Germany
[3] College of Food Science and Engineering, Northwest University, Xi'an 710000, China
* Correspondence: libinglin@nwu.edu.cn (B.L.); martin.gand@lcb.chemie.uni-giessen.de (M.G.)

Table S1. The primers used in this study to generate the different mutants. fw = forward, rv = reverse.

Name of the mutant	Orientation	Sequence (5'→3')
F91G	fw	GAATACCGTTGGCAAAGCCGTGTTTCCGG
	rv	CACGGCTTTGCCAACGGTATTCACCAGCAG
F91H	fw	GAATACCGTTCATAAAGCCGTGTTTCCGG
	rv	CACGGCTTTATGAACGGTATTCACCAGCAG
F91L	fw	GAATACCGTTCTGAAAGCCGTGTTTCCGG
	rv	CACGGCTTTCAGAACGGTATTCACCAGCAG
F91N	fw	GAATACCGTTAACAAAGCCGTGTTTCCGG
	rv	CACGGCTTTGTTAACGGTATTCACCAGCA
F91T	fw	GAATACCGTTACCAAAGCCGTGTTTCCGG
	rv	CACGGCTTTGGTAACGGTATTCACCAGCAG
F129A	fw	GGCGGCGCGGAACTGGGTAGTACCAGCATG
	rv	CCAGTTCCGCGCCGCCGCCAAAAATCCACACC
F129C	fw	GGCGGCTGCGAACTGGGTAGTACCAGCATG
	rv	CCAGTTCGCAGCCGCCGCCAAAAATCCACACC
F129M	fw	GGCGGCATGGAACTGGGTAGTACCAGCATG
	rv	CCAGTTCCATGCCGCCGCCAAAAATCCACACC

Name of the mutant	Orientation	Sequence (5′→3′)
F129R	fw	GGCGGCCGCGAACTGGGTAGTACCAGCATG
	rv	CCAGTTCGCGGCCGCCGCCAAAAATCCACACC
F129Q	fw	GGCGGCCAGGAACTGGGTAGTACCAGCATG
	rv	CCAGTTCCTGGCCGCCGCCAAAAATCCACACC
S163H	fw	GAATTATCGTCTGCATGGTTTTGGCTTTCTGGC
	rv	GCCAAAACCATGCAGACGATAATTCATGCTAAC
S163M	fw	GAATTATCGTCTGATGGGTTTTGGCTTTCTGGC
	rv	GCCAAAACCCATCAGACGATAATTCATGCTAAC
S163P	fw	GAATTATCGTCTGCCGGGTTTTGGCTTTCTGGC
	rv	GCCAAAACCCGGCAGACGATAATTCATGCTAAC
S163V	fw	GAATTATCGTCTGGTGGGTTTTGGCTTTCTGGC
	rv	GCCAAAACCCACCAGACGATAATTCATGCTAAC
S163Y	fw	GAATTATCGTCTGTACGGTTTTGGCTTTCTGGC
	rv	GCCAAAACCGTACAGACGATAATTCATGCTAAC
I245F	fw	GCAGAGTGGTAGCCCGTTTCCGGTGGGCG
	rv	GTAATATCGCCCACCGGAAACGGGCTACCAC
I245W	fw	CTTTATGCAGAGTGGTAGCCCGATTCCGGTG
	rv	CATGGGTAATATCGCCCACCGGAATCGGGC
L300R	fw	CCGTTTATTTTTGATTACCAGAGCCGTGTTCTGG
	rv	CGGCAGCCATGCCAGAACACGGCTCTGG
L300P	fw	CCGTTTATTTTTGATTACCAGAGCCCGGTTCTGG
	rv	CGGCAGCCATGCCAGAACCGGGCTCTGG
L300I	fw	CCGTTTATTTTTGATTACCAGAGCATTGTTCTGG
	rv	CGGCAGCCATGCCAGAACAATGCTCTGG
L300Q	fw	CCCGTTTATTTTTGATTACCAGAGCCTGGTTCTGGC
	rv	GTACGCGGCAGCCATGCCAGAACCAGGCTCTGG

Name of the mutant	Orientation	Sequence (5′→3′)
L302F	fw	GAGCCTGGTTTTCGCATGGCTGCCGCGTAC
	rv	GCAGCCATGCGAAAACCAGGCTCTGGTAATC
L302G	fw	GAGCCTGGTTGGCGCATGGCTGCCGCGTAC
	rv	GCAGCCATGCGCCAACCAGGCTCTGGTAATC
L302P	fw	GAGCCTGGTTCCGGCATGGCTGCCGCGTAC
	rv	GCAGCCATGCCGGAACCAGGCTCTGGTAATC
L305A	fw	CTGGTTCTGGCATGGGCGCCCGTAC
	rv	GAAAGGTACCATCTGTACGCGGCGCCCATG
L305M	fw	CTGGTTCTGGCATGGATGCCGCGTACAG
	rv	GAAAGGTACCATCTGTACGCGGCATCCATGCC
L305N	fw	CTGGTTCTGGCATGGAACCCGCGTACAG
	rv	GAAAGGTACCATCTGTACGCGGGTTCCATGCC
L305R	fw	CTGGTTCTGGCATGGCGTCCGCGTACAG
	rv	GAAAGGTACCATCTGTACGCGGACGCCATGCC
L305H	fw	CTGGTTCTGGCATGGCATCCGCGTACAG
	rv	GAAAGGTACCATCTGTACGCGGATGCCATGCC
L305Y	fw	CTGGTTCTGGCATGGTATCCGCGTACAG
	rv	GAAAGGTACCATCTGTACGCGGATACCATGCC
I529A	fw	CAAATCCGGCGTAAGCGGCCGCACTCGAGCAC
	rv	CCGCTTACGCCGGATTTGCCAGGGTCAGGCC
I529D	fw	CAAATCCGGATTAAGCGGCCGCACTCGAGCAC
	rv	CCGCTTAATCCGGATTTGCCAGGGTCAGGCC
I529E	fw	CAAATCCGGAATAAGCGGCCGCACTCGAGCAC
	rv	CCGCTTATTCCGGATTTGCCAGGGTCAGGCC
I529G	fw	CAAATCCGGGCTAAGCGGCCGCACTCGAGCAC
	rv	CCGCTTAGCCCGGATTTGCCAGGGTCAGGCC
I529W	fw	GACCCTGGCAAATCCGTGGTAAGCGGCCGC

Name of the mutant	Orientation	Sequence (5′→3′)
	rv	CTCGAGTGCGGCCGCTTACCACGGATTTGC

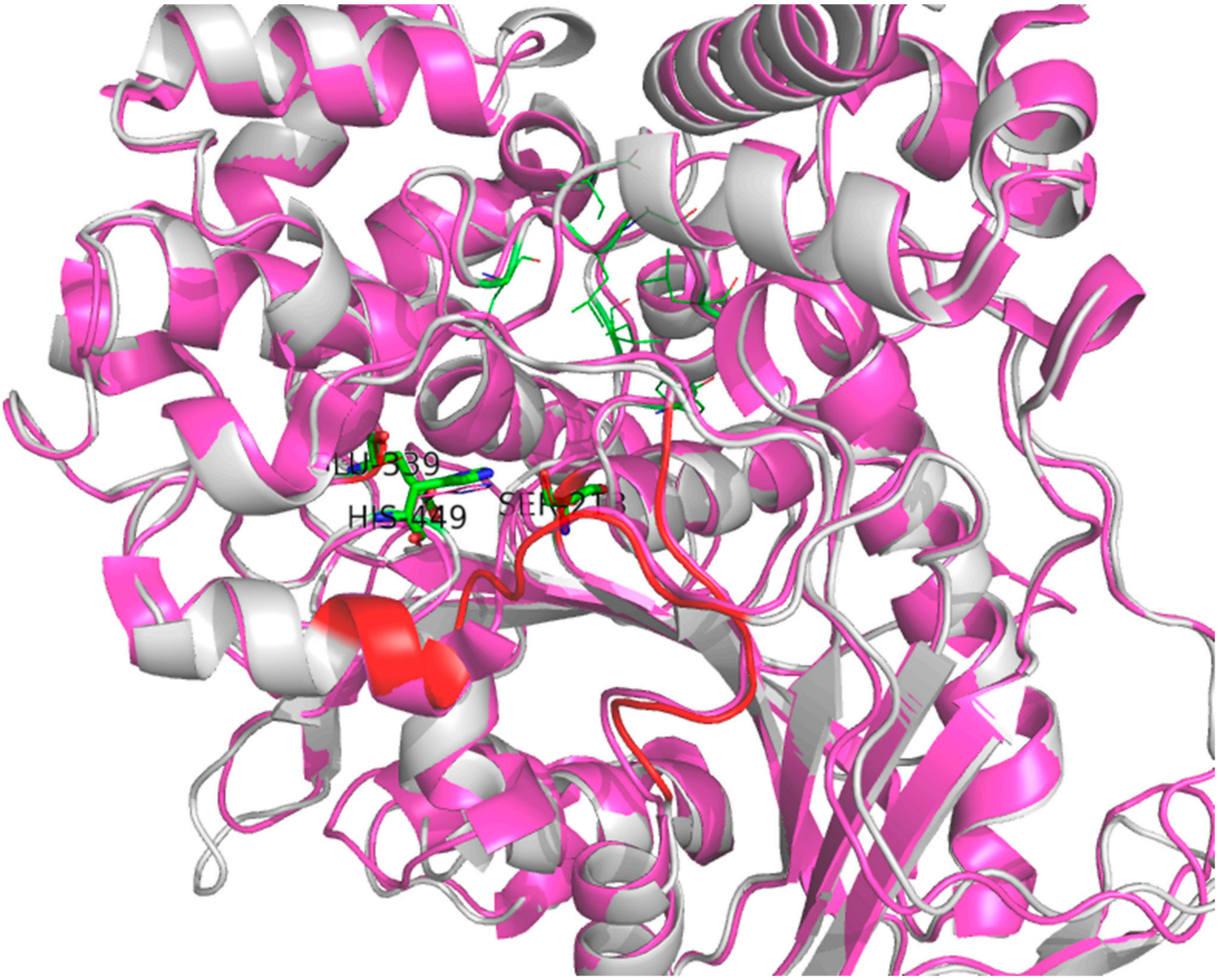

Figure S1. Structural alignment of the homology model of the PCI_Lip (grey) and *Candida rugosa* lipase 2 (PDB ID 1GZ7, magenta). The carbon atoms of the catalytic triad are colored in green for the PCI or in magenta for the 1GZ7, the nitrogen atoms are blue and the oxygen atoms are colored in red. The backbone of the entrance of the active pocket is marked in red.

```
PCI_8383/PCI_Lip,_P._citrinopileatus      MNPLPRTTSVTLDSASFTGSTIGRVTKFLGIPYAQPPTGDRRFRLPAPIPAYTGTVRATS  60
1GZ7_1|Chains                             -----APTATLANGDTITGLNAIVNEKFLGIPFAEPPVGTLRFKPPVPYSASLNGQQFTS  55
                                                *:.  :. ::** .     ******:*:**.*  **: *.*  *   .  : **

PCI_8383/PCI_Lip,_P._citrinopileatus      YGPSCPQQAVSLPLPDGLAADVADL-LVNTVFKAVFPDSEDCLTLNVVVPNSATPTSKLP  119
1GZ7_1|Chains                             YGPSCMQMNPMGSFEDTLPKNALDLVLQSKIFQVVLPNDEDCLTINVIRPPGTRASAGLP  115
                                          ***** *       : * *  :. ** * ..:*:.*:*:.*****:**: * .:  :: **

PCI_8383/PCI_Lip,_P._citrinopileatus      VVVWIFGGGFELGSTSMYDGGLIVERSIQLGEPVIYVSMNYRLSGFGFLASKEVKDAGVG  179
1GZ7_1|Chains                             VMLWIFGGGFELGGSSLFPGDQMVAKSVLMGKPVIHVSMNYRVASWGFLAGPDIQNEGSG  175
                                          *::***********.:*:: *. :* :*: :*:***:******::.:****. :::: * *

PCI_8383/PCI_Lip,_P._citrinopileatus      NLGLQDQREALRWIQKYIGNFGGDPTKVTIWGESAGAISAALHMVANNGN----TEGLFR  235
1GZ7_1|Chains                             NAGLHDQRLAMQWVADNIAGFGGDPSKVTIYGESAGSMSTFVHLVWNDGDNTYNGKPLFR  235
                                          * **:*** *::*: . *..*****:****:*****::*: :*:* *:*:      : ***

PCI_8383/PCI_Lip,_P._citrinopileatus      GAFMQSGSPIPVGDI--THGQATYDAIVRDTGCAGSSDTLACLRAAPYSALKNAIDNTPF  293
1GZ7_1|Chains                             AAIMQSGCMVPSDPVDGTYGTEIYNQVVASAGCGSASDKLACLRGLSQDTLYQATSDTPG  295
                                          .*:****. :* . :  *:*   *: :* .:**..:**.*****.    .:* :* .:**

PCI_8383/PCI_Lip,_P._citrinopileatus      IFDYQSLVLAWLPRTDGTFLTDNPQSLVQQGKVANVPFITGDCDDEGTLFSLANLNVTTT  353
1GZ7_1|Chains                             VLAYPSLRLSYLPRPDGTFITDDMYALVRDGKYAHVPVIIGDQNDEGTLFGLSSLNVTTD  355
                                          :: * ** *::*** ****:**:  :**::** *:**.* ** :******.*:.*****

PCI_8383/PCI_Lip,_P._citrinopileatus      SQLRTYLKTIWLPGTTDTQIDTLLSHYPLDLTQGSPYGTGILNALSPQFKRLASFQGDAV  413
1GZ7_1|Chains                             AQARAYFKQSFIH-ASDAEIDTLMAAYTSDITQGSPFDTGIFNAITPQFKRISALLGDLA  414
                                          :* *:*:*  ::  ::*::****:: *  *:*****:.***:**::*****:::: ** .

PCI_8383/PCI_Lip,_P._citrinopileatus      FQAPRRYFLQQRSGKQNTWAFLSKRFKVAPFLGSFHASDILNVYFGGE-----LGDYLIN  468
1GZ7_1|Chains                             FTLARRYFLNYYQGG-TKYSFLSKQLSGLPVLGTFHGNDIIWQDYLVGSGSVIYNNAFIA  473
                                          *   *****:  .*  ..::****::.  *.**:**..**:   :           .: :*

PCI_8383/PCI_Lip,_P._citrinopileatus      FVNKLDPNGAGRGINWPKYTTSSPNLVTFNDNLLFPVTLSQDTFRRDAINFLTGLTLANP  528
1GZ7_1|Chains                             FANDLDPNKAGLWTNWPTYTSSSQSGNNLMQINGLGLYTGKDNFRPDAYSALF----SNP  529
                                          *.*.**** **   ***.**:** .  .: :   : :  .:*.** ** . *     :**

PCI_8383/PCI_Lip,_P._citrinopileatus      I---- 529
1GZ7_1|Chains                             PSFFV 534
```

Figure S2. Sequence alignment of the PCI_Lip and *Candida rugosa* lipase 2 (PDB ID 1GZ7), the catalytic triad was confirmed as S213, E339 and H449 (numbering according to the PCI_Lip), which corresponds to the residues S209 (red box), E341 (blue box) and His449 (magenta box) and the oxyanion hole is marked in green box (G126-G128).

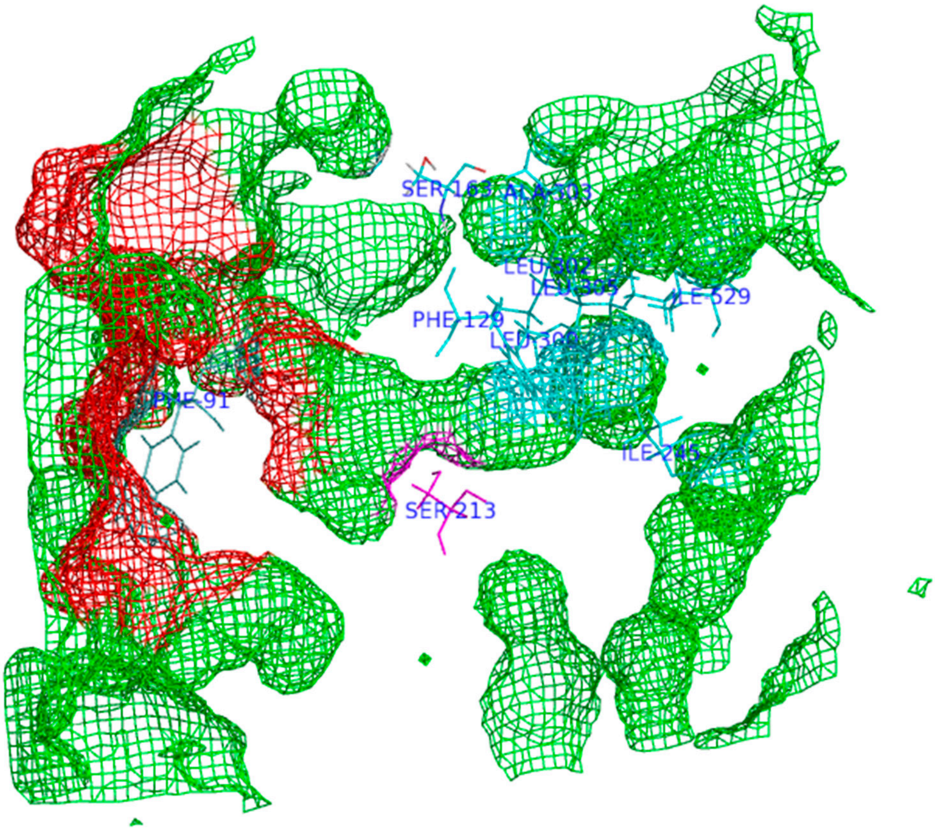

Figure S3. Analysis of the substrate tunnel by displaying the cavities around the nucleophilic S213 (marked in magenta), whereas the lid regions are marked in red. Tunnel restricting residues are colored in cyan. F91 is a special residues, as it is inside the lid region as well as forming part of the substrate entry tunnel.

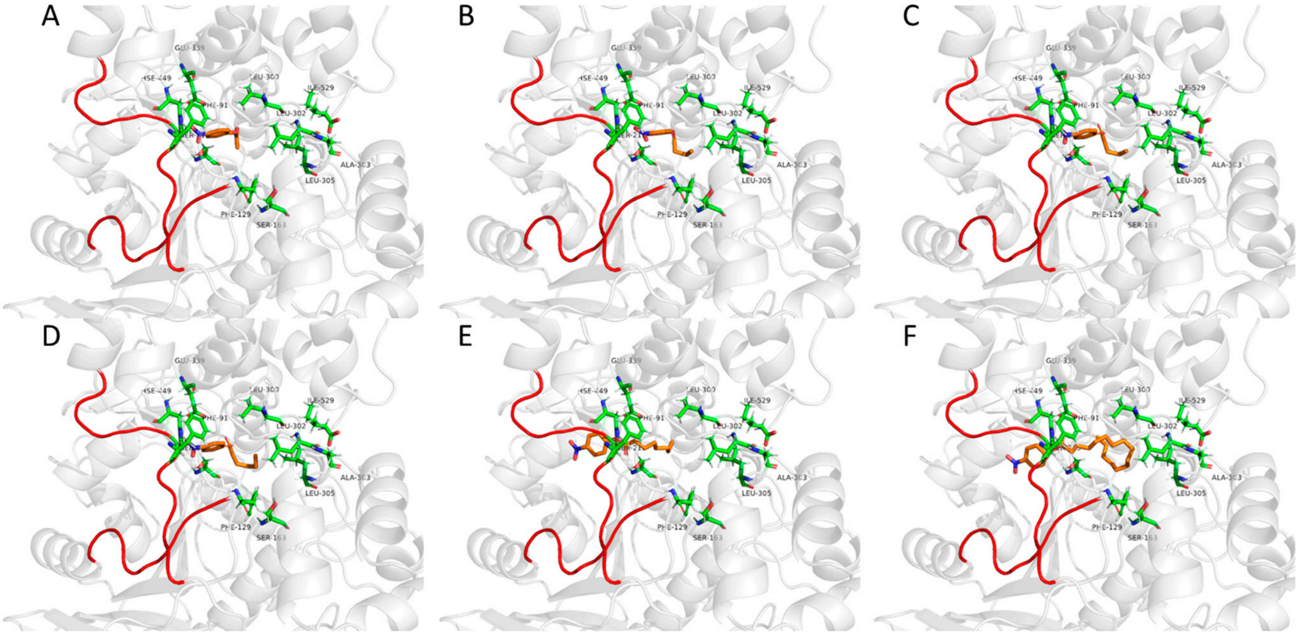

Figure S4. Docking of different chain length *p*-nitrophenol (pNP) esters inside the homology model of the PCI_Lip. Only the best docking results are shown. The criteria of a meaningful docking result is the distance of the S213 towards the carboxyl carbon atom, which were set to be < 5.1 Å for pNPA, 4.9 Å for pNPB, 4.6 Å for pNPV, 4.5 Å for pNPH, 4.5 Å for pNPO Å and 4.3 Å for pNPP. The carbon

atoms of the protein are colored in green, the nitrogen atoms are blue and the oxygen atoms are colored in red. The carbon atoms of the *p*-nitrophenol (pNP) esters are colored in orange. (**A**) pNP-acetate (pNPA), (**B**) pNP-butyrate (pNPB), (**C**) pNP-valerate (pNPV), (**D**) pNP-hexanoate (pNPH), (**E**) pNP-octanoate (pNPO), and (**F**) pNP-palmitate (pNPP). HSE = prototropic tautomer of histidine, H on N2 in the histidine ring, according to the CHARMM force field.

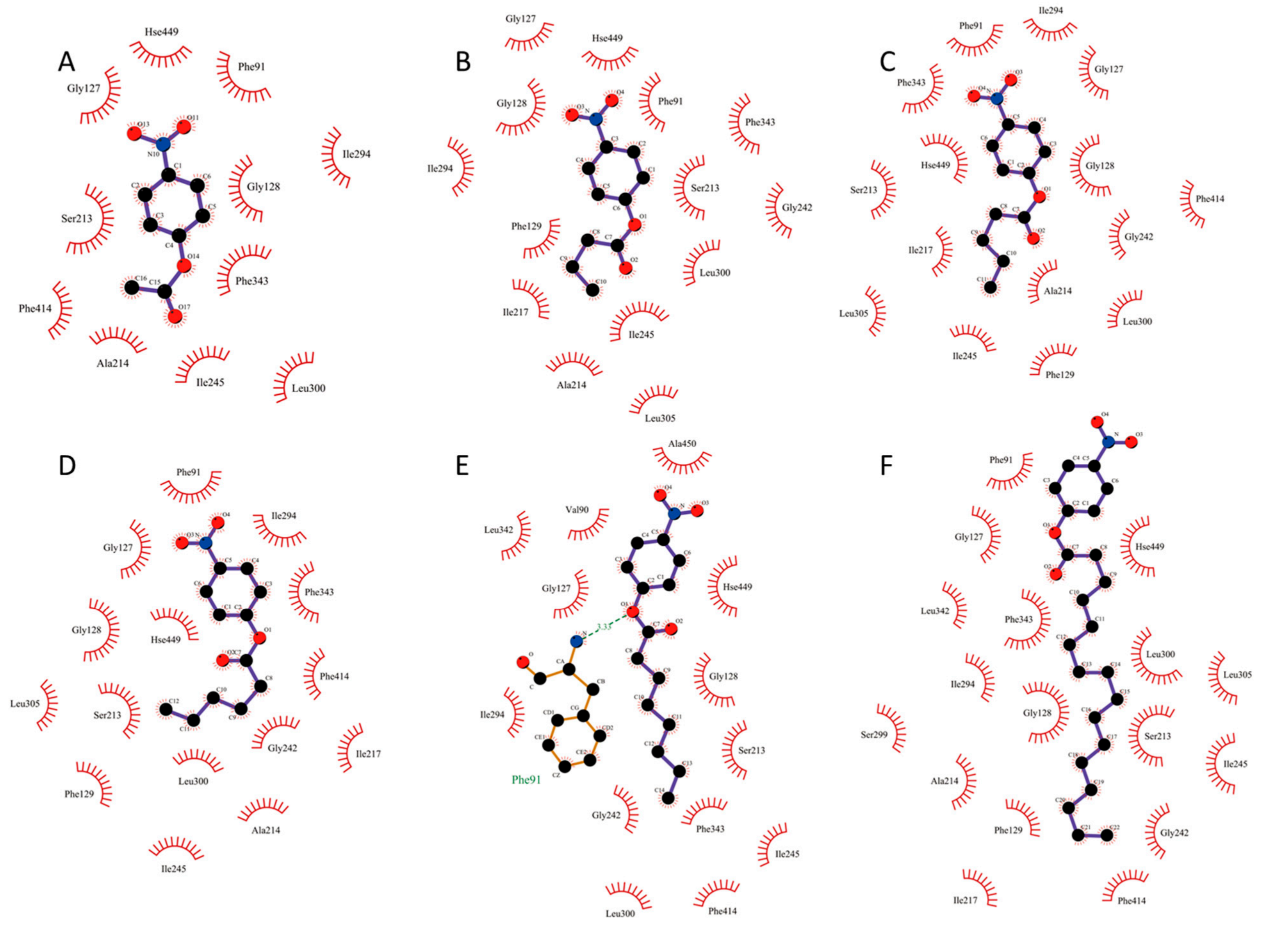

Figure S5. 2D pattern analysis of the docking of different chain length *p*-nitrophenol (pNP) esters inside the homology model of the PCI_Lip generated by using the software LigPloT. Only the best docking results are shown. The residues of the protein which are in the interacting distance are marked with red half circles. The carbon atoms of the *p*-nitrophenol (pNP) esters are colored in black and the bonds in magenta, the nitrogen atoms are blue and the oxygen atoms are colored in red. (**A**) pNP-acetate (pNPA), (**B**) pNP-butyrate (pNPB), (**C**) pNP-valerate (pNPV), (**D**) pNP-hexanoate (pNPH), (**E**) pNP-octanoate (pNPO), and (**F**) pNP-palmitate (pNPP). HSE = prototropic tautomer of histidine, H on N2 in the histidine ring, according to the CHARMM force field.

Table S2. Analysis of the free binding energy from the PCI_Lip WT and all exchanges at position F91 for the docking results of pNPA. The empty unit means that substrates couldn't be docked into the active pocket. Affinity at best position means the docking affinity at the minimum distance.

Mutant	Number of meaningful docking results	Minimum distance [Å]	Affinity at best position [kcal/mol]	Highest affinity [kcal/mol]
F91	2	5.1	−4.4	−4.4
F91G	5	3.3	−4.9	−5.3
F91P				
F91L	3	5.7	−5.8	−6
F91A	3	5.2	−4.9	−5.4
F91D				
F91E				
F91I	2	3.7	−4.9	−5
F91M				
F91N	2	4.4	−5.8	−5.8
F91Q				
F91R				
F91S				
F91V	1	3.7	−4.5	−4.5
F91W				
F91Y	1	6.1	−3.9	−3.9
F91K				
F91H	3	3.4	−5.8	−5.8
F91C				
F91T	2	5.1	−4.4	−4.4

Table S3. Analysis of the free binding energy from the PCI_Lip WT and all exchanges at position F91 for the docking results of pNPB. The empty unit means that substrates couldn't be docked into the active pocket. Affinity at best position means the docking affinity at the minimum distance.

Mutant	Number of meaningful docking results	Minimum distance [Å]	Affinity at best position [kcal/mol]	Highest affinity [kcal/mol]
F91	3	4.9	−4.8	−5.1
F91G	7	3.3	−5.4	−5.9
F91P				
F91L	5	3.9	−5.8	−6.5
F91A	1	3.4	−6.1	−6.1
F91D				
F91E	2	5	−3.7	−4.6
F91I	4	4.4	−5.7	−6.3
F91M				
F91N	5	3.5	−5.6	−5.9
F91Q	1	3.5	−6.5	−6.5

F91R				
F91S	3	5.2	−4.8	−5.3
F91V	1	6.1	−4.8	−4.8
F91W				
F91Y	2	5.8	−4.8	−4.9
F91K				
F91H	8	3.6	−6	−6.1
F91C				
F91T				

Table S4. Analysis of the free binding energy from the PCI_Lip WT and all exchanges at position F91 for the docking results of pNPV. The empty unit means that substrates couldn't be docked into the active pocket. Affinity at best position means the docking affinity at the minimum distance.

Mutant	Number of meaningful docking results	Minimum distance [Å]	Affinity at best position [kcal/mol]	Highest affinity [kcal/mol]
F91	5	4.6	−5.1	−5.1
F91G	4	3.5	−5	−5.9
F91P				
F91L	9	3.9	−5.7	−6.8
F91A				
F91D				
F91E	3	3.5	−5.6	−6
F91I	2	5	−6.1	−6.1
F91M	1	5.8	−5.8	−5.8
F91N	6	3.3	−5.3	−6.2
F91Q				
F91R				
F91S	3	4	−5.2	−5.6
F91V				
F91W				
F91Y	6	5.6	−5.7	−6
F91K				
F91H	6	3.6	−5.8	−6.1
F91C				
F91T	2	2.7	−5.6	−5.6

Table S5. Analysis of the free binding energy from the PCI_Lip WT and all exchanges at position F91 for the docking results of pNPH. The empty unit means that substrates couldn't be docked into the active pocket. Affinity at best position means the docking affinity at the minimum distance.

Mutant	Number of meaningful docking results	Minimum distance [Å]	Affinity at best position [kcal/mol]	Highest affinity [kcal/mol]
F91	3	4.5	−5.8	−5.9
F91G	7	3.2	−5.4	−5.4
F91P				
F91L	8	3.7	−6.1	−7
F91A	2	3.3	−4.6	−5.7
F91D				
F91E	2	3.4	−5	−5
F91I	4	5	−6	−6.7
F91M				
F91N	5	3.5	−6.4	−6.4
F91Q				
F91R				
F91S	1	5.3	−5.2	−5.2
F91V				
F91W				
F91Y	3	5.5	−5.1	−5.1
F91K				
F91H	8	3.8	−5.4	−5.7
F91C				
F91T	3	2.9	−5.7	−5.7

Table S6. Analysis of the free binding energy from the PCI_Lip WT and all exchanges at position F91 for the docking results of pNPO. The empty unit means that substrates couldn't be docked into the active pocket. Affinity at best position means the docking affinity at the minimum distance.

Mutant	Number of meaningful docking results	Minimum distance [Å]	Affinity at best position [kcal/mol]	Highest affinity [kcal/mol]
F91	5	4.5	−5.8	−5.9
F91G	5	2.7	−4	−5.6
F91P				
F91L	8	4.5	−6.2	−7.6
F91A	3	3.4	−5.8	−5.8
F91D				
F91E	4	3.4	−5.8	−6.1
F91I	6	4.6	−5.6	−6.4
F91M				
F91N	4	3.9	−6	−6.8
F91Q				

F91R				
F91S	3	4.9	−4.6	−5.1
F91V				
F91W				
F91Y	5	4.5	−4.9	−6
F91K				
F91H	11	3.6	−5.4	−6.3
F91C				
F91T	3	2.7	−5.8	−6.3

Table S7. Analysis of the free binding energy from the PCI_Lip WT and all exchanges at position F91 for the docking results of pNPP. The empty unit means that substrates couldn't be docked into the active pocket. Affinity at best position means the docking affinity at the minimum distance.

Mutant	Number of meaningful docking results	Minimum distance [Å]	Affinity at best position [kcal/mol]	Highest affinity [kcal/mol]
F91	6	4.3	−4.2	−5.5
F91G	4	2.9	−3.7	−3.7
F91P				
F91L	13	3.6	−4.6	−4.6
F91A	4	3.5	−6.1	−6.1
F91D				
F91E	3	3.8	−6.7	−6.7
F91I	7	3.7	−6.8	−7
F91M	1	5.9	−7.5	−7.5
F91N	3	3.9	−7.1	−7.1
F91Q				
F91R				
F91S				
F91V	1	3.9	−5.7	−5.7
F91W				
F91Y	7	2.8	−5.4	−6.1
F91K				
F91H	6	3.4	−6.6	−6.6
F91C				
F91T	3	2.7	−6.8	−6.8

Table S8. Analysis of the free binding energy from the PCI_Lip WT and all exchanges at position L302 for the docking results of pNPA. The empty unit means that substrates couldn't be docked into the active pocket. Affinity at best position means the docking affinity at the minimum distance.

Mutant	Number of meaningful docking results	Minimum distance [Å]	Affinity at best position [kcal/mol]	Highest affinity [kcal/mol]
L302	2	5.1	−4.4	−4.4
L302G	5	3.7	−6.7	−6.7
L302P	5	3.9	−5.1	−5.5
L302F	1	3.9	−2.8	−2.8
L302A				
L302D	1	6.2	−5.2	−5.2
L302E				
L302I				
L302M				
L302N				
L302Q	6	4.9	−4.8	−5.6
L302R				
L302S				
L302V				
L302W				
L302Y	2	4.7	−5.5	−5.5
L302K	1	4	−4.5	−4.5
L302H	1	6.4	−4.2	−4.2
L302C				
L302T				

Table S9. Analysis of the free binding energy from the PCI_Lip WT and all exchanges at position L302 for the docking results of pNPB. The empty unit means that substrates couldn't be docked into the active pocket. Affinity at best position means the docking affinity at the minimum distance.

Mutant	Number of meaningful docking results	Minimum distance [Å]	Affinity at best position [kcal/mol]	Highest affinity [kcal/mol]
L302	3	4.9	−4.8	−5.1
L302G	8	3.3	−6.8	−7.1
L302P	6	3.9	−5.7	−5.8
L302F	1	4.1	−3.5	−3.5
L302A				
L302D	3	4.2	−5.5	−5.5
L302E				
L302I				
L302M				
L302N	4	5.8	−6.2	−6.3
L302Q	4	3.4	−5.7	−6.2

L302R				
L302S				
L302V	3	5.4	−3.7	−3.7
L302W	4	4.9	1.1	0.1
L302Y	2	5.1	−5.9	−6.1
L302K	1	5	−4.9	−4.9
L302H	1	6.4	−4.6	−4.6
L302C				
L302T				

Table S10. Analysis of the free binding energy from the PCI_Lip WT and all exchanges at position L302 for the docking results of pNPV. The empty unit means that substrates couldn't be docked into the active pocket. Affinity at best position means the docking affinity at the minimum distance.

Mutant	Number of meaningful docking results	Minimum distance [Å]	Affinity at best position [kcal/mol]	Highest affinity [kcal/mol]
L302	5	4.6	−5.1	−5.1
L302G	8	3.3	−7.2	−7.2
L302P	6	5.3	−5.5	−5.8
L302F	1	5.2	−3.7	−3.7
L302A				
L302D	2	6	−5.5	−5.5
L302E	1	5	−2.7	−2.7
L302I				
L302M				
L302N	1	5.8	−5.9	−5.9
L302Q	4	4	−5.7	−6.4
L302R				
L302S				
L302V	1	6.5	−4.5	−4.5
L302W				
L302Y				
L302K				
L302H				
L302C				
L302T				

Table S11. Analysis of the free binding energy from the PCI_Lip WT and all exchanges at position L302 for the docking results of pNPH. The empty unit means that substrates couldn't be docked into the active pocket. Affinity at best position means the docking affinity at the minimum distance.

Mutant	Number of meaningful docking results	Minimum distance [Å]	Affinity at best position [kcal/mol]	Highest affinity [kcal/mol]
L302	3	4.5	−5.8	−5.9
L302G	10	3.3	−7.1	−7.3
L302P	7	4.4	−5.5	−5.9
L302F	3	4.1	−3.8	−5
L302A				
L302D	3	4.3	−5.9	−5.9
L302E				
L302I				
L302M				
L302N	3	5.3	−6.4	−6.4
L302Q	4	5.2	−6.1	−6.2
L302R				
L302S				
L302V	3	5.3	−4.1	−4.8
L302W				
L302Y	1	5.4	−4.9	−4.9
L302K				
L302H				
L302C				
L302T				

Table S12. Analysis of the free binding energy from the PCI_Lip WT and all exchanges at position L302 for the docking results of pNPO. The empty unit means that substrates couldn't be docked into the active pocket. Affinity at best position means the docking affinity at the minimum distance.

Mutant	Number of meaningful docking results	Minimum distance [Å]	Affinity at best position [kcal/mol]	Highest affinity [kcal/mol]
L302	5	4.5	−5.8	−5.9
L302G	12	2.8	−5.2	−7.8
L302P	11	4	−5.3	−5.9
L302F	4	4.3	−4	−4.5
L302A				
L302D	2	4.7	−6	−6
L302E	1	4.5	−3.1	−3.1
L302I				
L302M				
L302N	2	6.1	−5.8	−6.4
L302Q	7	5.5	−5.7	−5.8

L302R				
L302S				
L302V	1	6.3	−5.2	−5.2
L302W				
L302Y	2	4.9	−5.5	−5.5
L302K				
L302H				
L302C				
L302T				

Table S13. Analysis of the free binding energy from the PCI_Lip WT and all exchanges at position L302 for the docking results of pNPP. The empty unit means that substrates couldn't be docked into the active pocket. Affinity at best position means the docking affinity at the minimum distance.

Mutant	Number of meaningful docking results	Minimum distance [Å]	Affinity at best position [kcal/mol]	Highest affinity [kcal/mol]
L302	6	4.3	−4.2	−5.5
L302G	8	3.7	−6.6	−7.7
L302P	7	3.9	−6.4	−7.6
L302F				
L302A				
L302D	3	5.1	−6.5	−6.5
L302E				
L302I				
L302M				
L302N	2	5.3	−6.5	−6.5
L302Q	10	3.4	−6.7	−7
L302R				
L302S				
L302V	2	6.1	−3.7	−4.9
L302W	4	5.6	5.1	5.1
L302Y	1	5.5	−0.6	−0.6
L302K				
L302H				
L302C				
L302T				

Table S14. Analysis of the free binding energy from the PCI_Lip WT and all exchanges at position L300 for the docking results of pNPA. The empty unit means that substrates couldn't be docked into the active pocket. Affinity at best position means the docking affinity at the minimum distance.

Mutant	Number of meaningful docking results	Minimum distance [Å]	Affinity at best position [kcal/mol]	Highest affinity [kcal/mol]
L300	2	5.1	−4.4	−4.4
L300G				
L300P	6	3.4	−5.8	−6.4
L300F	3	5.7	−5.8	−6
L300A	2	3.8	−5.9	−5.9
L300D				
L300E				
L300I	5	4.4	−5.2	−6
L300M	7	4.9	−6	−6
L300N	1	5.4	−2.8	−2.8
L300Q	3	4.5	−5.6	−5.6
L300R	5	4.9	−4.7	−6.4
L300S				
L300V				
L300W				
L300Y				
L300K				
L300H				
L300C				
L300T				

Table S15. Analysis of the free binding energy from the PCI_Lip WT and all exchanges at position L300 for the docking results of pNPB. The empty unit means that substrates couldn't be docked into the active pocket. Affinity at best position means the docking affinity at the minimum distance.

Mutant	Number of meaningful docking results	Minimum distance [Å]	Affinity at best position [kcal/mol]	Highest affinity [kcal/mol]
L300	3	4.9	−4.8	−5.1
L300G				
L300P	4	4.2	−6.6	−6.6
L300F	5	3.9	−5.8	−6.5
L300A	4	3.7	−5.8	−5.9
L300D	2	5.1	−4.9	−5.1
L300E				
L300I	5	5.1	−5.9	−6.1
L300M	6	5.5	−5.5	−6.6
L300N	6	3.5	−2.1	−4.7
L300Q	3	3.8	−6.2	−6.3

L300R	6	4.1	−6.2	−7
L300S	1	5.9	−1.9	−1.9
L300V				
L300W				
L300Y				
L300K				
L300H				
L300C				
L300T	1	6.2	−5	−5

Table S16. Analysis of the free binding energy from the PCI_Lip WT and all exchanges at position L300 for the docking results of pNPV. The empty unit means that substrates couldn't be docked into the active pocket. Affinity at best position means the docking affinity at the minimum distance.

Mutant	Number of meaningful docking results	Minimum distance [Å]	Affinity at best position [kcal/mol]	Highest affinity [kcal/mol]
L300	5	4.6	−5.1	−5.1
L300G				
L300P	3	3.8	−6.4	−6.7
L300F	9	3.9	−5.7	−6.8
L300A	3	3.2	−5.5	−6.2
L300D	1	5.7	−5.5	−5.5
L300E	4	3.4	−2.7	−4.5
L300I	6	5	−6	−6.2
L300M	5	5.7	−6.5	−6.5
L300N	1	6.3	−3.9	−3.9
L300Q	4	4.1	−5.7	−6.2
L300R	6	4.1	−6.3	−7.1
L300S	2	5.5	−2.1	−2.2
L300V				
L300W				
L300Y				
L300K	1	5.6	−5.7	−5.7
L300H				
L300C				
L300T				

Table S17. Analysis of the free binding energy from the PCI_Lip WT and all exchanges at position L300 for the docking results of pNPH. The empty unit means that substrates couldn't be docked into the active pocket. Affinity at best position means the docking affinity at the minimum distance.

Mutant	Number of meaningful docking results	Minimum distance [Å]	Affinity at best position [kcal/mol]	Highest affinity [kcal/mol]
L300	3	4.5	−5.8	−5.9
L300G				
L300P	9	3.5	−6.9	−7.3
L300F	8	3.7	−6.1	−7
L300A	2	5.8	−5.5	−5.5
L300D	2	6.2	−5.2	−5.2
L300E	3	3.6	−2.6	−5.7
L300I	11	4.6	−5.7	−6.5
L300M	5	5.2	−5.8	−6.8
L300N	3	3.4	−2.2	−3.8
L300Q	1	3.9	−6.1	−6.1
L300R	9	4.2	−6.4	−7.1
L300S				
L300V				
L300W				
L300Y				
L300K				
L300H				
L300C				
L300T				

Table S18. Analysis of the free binding energy from the PCI_Lip WT and all exchanges at position L300 for the docking results of pNPO. The empty unit means that substrates couldn't be docked into the active pocket. Affinity at best position means the docking affinity at the minimum distance.

Mutant	Number of meaningful docking results	Minimum distance [Å]	Affinity at best position [kcal/mol]	Highest affinity [kcal/mol]
L300	5	4.5	−5.8	−5.9
L300G				
L300P	8	3.6	−7	−7.3
L300F	8	4.5	−6.2	−7.6
L300A	1	5.9	−3.5	−3.5
L300D	2	4.1	−5.4	−5.4
L300E				
L300I	9	4.4	−6.2	−6.9
L300M	3	4.6	−4.6	−6.9
L300N	1	6.2	−1.9	−1.9
L300Q	4	3.5	−4.7	−6.1

L300R	5	4	−6.4	−7.3
L300S	1	5.6	−1.1	−1.1
L300V				
L300W				
L300Y				
L300K				
L300H				
L300C				
L300T				

Table S19. Analysis of the free binding energy from the PCI_Lip WT and all exchanges at position L300 for the docking results of pNPP. The empty unit means that substrates couldn't be docked into the active pocket. Affinity at best position means the docking affinity at the minimum distance.

Mutant	Number of meaningful docking results	Minimum distance [Å]	Affinity at best position [kcal/mol]	Highest affinity [kcal/mol]
L300	6	4.3	−4.2	−5.5
L300G				
L300P	9	4.1	−5.8	−7.7
L300F	13	3.6	−4.6	−4.6
L300A	4	3.7	−6.8	−7.5
L300D	1	4	−6.1	−6.1
L300E	3	5.3	−3	−3.4
L300I	6	4.6	−5.7	−6.1
L300M	2	5.5	−5.6	−6.5
L300N				
L300Q				
L300R	7	5.8	−5.7	−6.2
L300S	3	4.8	1.6	0.7
L300V				
L300W				
L300Y	1	6.4	−3.7	−3.7
L300K	2	3.4	−6	−6
L300H				
L300C				
L300T				

Table S20. Analysis of the free binding energy from the PCI_Lip WT and all exchanges at position L305 for the docking results of pNPA. The empty unit means that substrates couldn't be docked into the active pocket. Affinity at best position means the docking affinity at the minimum distance.

Mutant	Number of meaningful docking results	Minimum distance [Å]	Affinity at best position [kcal/mol]	Highest affinity [kcal/mol]
L305	2	5.1	−4.4	−4.4
L305G				
L305P				
L305F				
L305A	3	4.1	−4.6	−4.9
L305D				
L305E				
L305I	2	5.2	−5.2	−5.5
L305M	4	4	−5.8	−5.8
L305N	3	4.3	−5.8	−5.8
L305Q	3	4.7	−0.3	−1.6
L305R	4	−1.3	−5.6	−5.6
L305S				
L305V				
L305W				
L305Y	4	4.1	−5	−5
L305K				
L305H	1	3.6	−4.7	−4.7
L305C				
L305T				

Table S21. Analysis of the free binding energy from the PCI_Lip WT and all exchanges at position L305 for the docking results of pNPB. The empty unit means that substrates couldn't be docked into the active pocket. Affinity at best position means the docking affinity at the minimum distance.

Mutant	Number of meaningful docking results	Minimum distance [Å]	Affinity at best position [kcal/mol]	Highest affinity [kcal/mol]
L305	3	4.9	−4.8	−5.1
L305G				
L305P				
L305F				
L305A	4	4.7	−5.2	−5.2
L305D				
L305E				
L305I	2	5	−3.7	−3.7
L305M	3	3.9	−5.9	−5.9
L305N	3	4.1	−5.7	−6.2
L305Q	2	5.9	−0.2	−0.2

L305R	2	4.8	−5.8	−5.8
L305S				
L305V				
L305W				
L305Y	3	3.8	−5.5	−5.5
L305K				
L305H	3	3.4	−5.5	−5.5
L305C				
L305T				

Table S22. Analysis of the free binding energy from the PCI_Lip WT and all exchanges at position L305 for the docking results of pNPV. The empty unit means that substrates couldn't be docked into the active pocket. Affinity at best position means the docking affinity at the minimum distance.

Mutant	Number of meaningful docking results	Minimum distance [Å]	Affinity at best position [kcal/mol]	Highest affinity [kcal/mol]
L305	5	4.6	−5.1	−5.1
L305G				
L305P				
L305F				
L305A	5	3.8	−6.8	−6.8
L305D				
L305E				
L305I	1	5.6	−4.6	−4.6
L305M	7	4.8	−4.8	−5.6
L305N	5	3.9	−5	−5
L305Q				
L305R	4	3.5	−4.9	−4.9
L305S				
L305V				
L305W				
L305Y	3	4.6	−5	−5.8
L305K				
L305H	3	4.6	−5	−5
L305C				
L305T				

Table S23. Analysis of the free binding energy from the PCI_Lip WT and all exchanges at position L305 for the docking results of pNPH. The empty unit means that substrates couldn't be docked into the active pocket. Affinity at best position means the docking affinity at the minimum distance.

Mutant	Number of meaningful docking results	Minimum distance [Å]	Affinity at best position [kcal/mol]	Highest affinity [kcal/mol]
L305	3	4.5	−5.8	−5.9
L305G				
L305P				
L305F				
L305A	5	3.9	−6.1	−6.1
L305D				
L305E				
L305I	1	6.5	−5.1	−5.1
L305M	8	4.9	−5.4	−5.7
L305N	7	4.3	−6	−6.7
L305Q				
L305R	6	3.5	−5.7	−5.8
L305S				
L305V				
L305W				
L305Y	2	4.5	−5.3	−5.6
L305K				
L305H	3	4.8	−5.2	−5.8
L305C	1	2.9	−3	−3
L305T				

Table S24. Analysis of the free binding energy from the PCI_Lip WT and all exchanges at position L305 for the docking results of pNPO. The empty unit means that substrates couldn't be docked into the active pocket. Affinity at best position means the docking affinity at the minimum distance.

Mutant	Number of meaningful docking results	Minimum distance [Å]	Affinity at best position [kcal/mol]	Highest affinity [kcal/mol]
L305	5	4.5	−5.8	−5.9
L305G	2	6.5	−1.4	−1.4
L305P				
L305F				
L305A	8	3.4	−5.6	−6.2
L305D				
L305E				
L305I	6	5.9	−2.5	−2.5
L305M	7	4.5	−6.1	−6.2
L305N	5	4.2	−6.3	−6.3
L305Q				

L305R	5	3.4	−6	−6.1
L305S				
L305V				
L305W				
L305Y	6	4.2	−6.2	−6.5
L305K				
L305H	7	3.3	−4.4	−4.6
L305C	3	3	3.7	3.7
L305T				

Table S25. Analysis of the free binding energy from the PCI_Lip WT and all exchanges at position L305 for the docking results of pNPP. The empty unit means that substrates couldn't be docked into the active pocket. Affinity at best position means the docking affinity at the minimum distance.

Mutant	Number of meaningful docking results	Minimum distance [Å]	Affinity at best position [kcal/mol]	Highest affinity [kcal/mol]
L305	6	4.3	−4.2	−5.5
L305G				
L305P				
L305F				
L305A				
L305D				
L305E				
L305I				
L305M	4	4	−6.4	−7.2
L305N	4	4.7	−6.7	−7
L305Q				
L305R	4	3.7	−6.3	−6.9
L305S				
L305V				
L305W				
L305Y	8	3.9	−6.8	−7
L305K				
L305H	4	3.4	−6	−6
L305C	1	4.3	1.3	1.3
L305T				

Table S26. Analysis of the free binding energy from the PCI_Lip WT and all exchanges at position I529 for the docking results of pNPA. The empty unit means that substrates couldn't be docked into the active pocket. Affinity at best position means the docking affinity at the minimum distance.

Mutant	Number of meaningful docking results	Minimum distance [Å]	Affinity at best position [kcal/mol]	Highest affinity [kcal/mol]
I529	2	5.1	−4.4	−4.4
I529G	3	5.7	−6	−6
I529P				
I529L	1	6.3	−5.4	−5.4
I529A	5	4.2	−5	−5.6
I529D				
I529E	1	4.8	−5.2	−5.2
I529F	1	4.9	−3.2	−3.2
I529M				
I529N				
I529Q				
I529R				
I529S	1	6.4	−5.3	−5.3
I529V				
I529W				
I529Y				
I529K	2	5.2	−5.8	−5.8
I529H				
I529C	4	5.4	−5.1	−5.6
I529T	1	6	−4.9	−4.9

Table S27. Analysis of the free binding energy from the PCI_Lip WT and all exchanges at position I529 for the docking results of pNPB. The empty unit means that substrates couldn't be docked into the active pocket. Affinity at best position means the docking affinity at the minimum distance.

Mutant	Number of meaningful docking results	Minimum distance [Å]	Affinity at best position [kcal/mol]	Highest affinity [kcal/mol]
I529	3	4.9	−4.8	−5.1
I529G	5	4	−5.8	−6.5
I529P				
I529L	2	5.8	−5.5	−6
I529A	6	4.9	−5.7	−6.2
I529D				
I529E	5	4	−5.8	−6.6
I529M				
I529N				
I529Q				
I529R				

I529S	1	6.5	−5.6	−5.6
I529V				
I529W				
I529Y	1	5.9	−4.3	−4.3
I529K	4	4.9	−6.3	−6.3
I529H				
I529C	4	4.9	−5.9	−6.9
I529T	1	5.1	−5	−5
I529F				

Table S28. Analysis of the free binding energy from the PCI_Lip WT and all exchanges at position I529 for the docking results of pNPV. The empty unit means that substrates couldn't be docked into the active pocket. Affinity at best position means the docking affinity at the minimum distance.

Mutant	Number of meaningful docking results	Minimum distance [Å]	Affinity at best position [kcal/mol]	Highest affinity [kcal/mol]
I529	5	4.6	−5.1	−5.1
I529G	4	3.4	−5.9	−6.7
I529P				
I529L	5	4.9	−3.9	−5.8
I529A	7	4.5	−5.7	−6.2
I529D				
I529E	8	4.2	−6.1	−6.8
I529M				
I529N				
I529Q				
I529R				
I529S				
I529V				
I529W				
I529Y	1	5.5	−4	−6.2
I529K				
I529H				
I529C	3	Affinity at best position [kcal/mol]	Highest affinity [kcal/mol]	−7.1
I529T	2	5.3	−5.5	−5.9
I529F				

Table S29. Analysis of the free binding energy from the PCI_Lip WT and all exchanges at position I529 for the docking results of pNPH. The empty unit means that substrates couldn't be docked into the active pocket. Affinity at best position means the docking affinity at the minimum distance.

Mutant	Number of meaningful docking results	Minimum distance [Å]	Affinity at best position [kcal/mol]	Highest affinity [kcal/mol]
I529	3	4.5	−5.8	−5.9
I529G	6	5.1	−5.9	−7
I529P				
I529L	5	4.8	−3.6	−6.1
I529A	8	3.3	−5.5	−6.4
I529D				
I529E	7	4	−6.1	−6.9
I529M				
I529N				
I529Q				
I529R				
I529S	1	6.3	−5.8	−5.8
I529V				
I529W				
I529Y	1	5.9	−5.2	−5.2
I529K	4	4.9	−6.2	−6.2
I529H				
I529C	2	5.7	−7.2	−7.2
I529T	2	5.3	−5.4	−5.4
I529F				

Table S30. Analysis of the free binding energy from the PCI_Lip WT and all exchanges at position I529 for the docking results of pNPO. The empty unit means that substrates couldn't be docked into the active pocket. Affinity at best position means the docking affinity at the minimum distance.

Mutant	Number of meaningful docking results	Minimum distance [Å]	Affinity at best position [kcal/mol]	Highest affinity [kcal/mol]
I529	5	4.5	−5.8	−5.9
I529G	6	3.9	−6.2	−7.2
I529P	4	4.2	−5.6	−5.6
I529L	7	3.1	−2.2	−5.3
I529A	4	4.3	−6.6	−6.6
I529D				
I529E	5	5	−5.7	−6.9
I529I	6	4.6	−5.6	−6.4
I529M				
I529N				
I529Q				

I529R	2	6.1	−5	−5.4
I529S	1	5.7	−5.8	−5.8
I529V				
I529W				
I529Y	2	5.6	−4.2	−5.3
I529K	6	4.9	−6.3	−6.3
I529H				
I529C	2	5.3	−7.1	−7.6
I529T	1	5.3	−6.8	−6.8

Table S31. Analysis of the free binding energy from the PCI_Lip WT and all exchanges at position I529 for the docking results of pNPP. The empty unit means that substrates couldn't be docked into the active pocket. Affinity at best position means the docking affinity at the minimum distance.

Mutant	Number of meaningful docking results	Minimum distance [Å]	Affinity at best position [kcal/mol]	Highest affinity [kcal/mol]
I529	6	4.3	−4.2	−5.5
I529G	8	5.7	−7.6	−7.6
I529P				
I529L	6	3.2	−2.3	−6.1
I529A	7	3.4	−5.2	−6.3
I529D				
I529E	10	3.8	−6.8	−7.5
I529M				
I529N				
I529Q				
I529R	5	4.1	−4.1	−5.9
I529S				
I529V				
I529W				
I529Y	5	5.8	−6.5	−6.8
I529K	7	5.1	−6.7	−6.7
I529H				
I529C	3	5	−6.7	−8.1
I529T	2	5.3	−6.8	−6.8
I529F				

Table S32. Analysis of the free binding energy from the PCI_Lip WT and all exchanges at position S163 for the docking results of pNPA. The empty unit means that substrates couldn't be docked into the active pocket. Affinity at best position means the docking affinity at the minimum distance.

Mutant	Number of meaningful docking results	Minimum distance [Å]	Affinity at best position [kcal/mol]	Highest affinity [kcal/mol]
S163	2	5.1	−4.4	−4.4
S163G				
S163P	1	4.9	−4.5	−4.5
S163L				
S163A				
S163D				
S163E	4	5.3	−5	−5.6
S163I				
S163M	2	4.7	−5	−5.6
S163N				
S163Q	2	4.3	−4.9	−4.9
S163R	1	4	−1.4	−1.4
S163F				
S163V	2	3.4	−5.9	−5.9
S163W	1	5.7	−3.9	−3.9
S163Y	1	5.7	−5.1	−5.1
S163K	2	6.2	−4.4	−4.4
S163H	4	4.2	−4.7	−6
S163C				
S163T				

Table S33. Analysis of the free binding energy from the PCI_Lip WT and all exchanges at position S163 for the docking results of pNPB. The empty unit means that substrates couldn't be docked into the active pocket. Affinity at best position means the docking affinity at the minimum distance.

Mutant	Number of meaningful docking results	Minimum distance [Å]	Affinity at best position [kcal/mol]	Highest affinity [kcal/mol]
S163	3	4.9	−4.8	−5.1
S163G				
S163P	3	4.7	−4.3	−4.4
S163L				
S163A				
S163D				
S163E	3	4	−5.6	−5.6
S163I				
S163M	4	4.5	−5.3	−6
S163N				
S163Q	3	4	−5.2	−5.2

S163R				
S163V	1	5.9	−1.9	−1.9
S163W	6	3.4	−6.2	−6.2
S163Y				
S163K	2	5.6	−5.2	−5.2
S163H	4	5.9	−3.6	−4.1
S163C	9	3.7	−4.8	−6.7
S163T	1	6.4	−3.2	−3.2
S163F	1	3.7	−2.2	−2.2

Table S34. Analysis of the free binding energy from the PCI_Lip WT and all exchanges at position S163 for the docking results of pNPV. The empty unit means that substrates couldn't be docked into the active pocket. Affinity at best position means the docking affinity at the minimum distance.

Mutant	Number of meaningful docking results	Minimum distance [Å]	Affinity at best position [kcal/mol]	Highest affinity [kcal/mol]
S163	5	4.6	−5.1	−5.1
S163G				
S163P	4	3.4	−5	−5
S163L				
S163A	2	4.5	−5.2	−5.2
S163D				
S163E	2	6	−5.7	−5.9
S163I	1	6.3	−5.7	−5.7
S163M	6	4.2	−5.9	−6.1
S163N				
S163Q	2	4.7	−5.6	−5.6
S163R				
S163V	2	5.5	−2.1	−2.2
S163W	1	5.8	−5.3	−5.3
S163Y				
S163K				
S163H	1	6.3	−2.9	−2.9
S163C	5	3.8	−5.7	−6.8
S163T				
S163F	1	6	−3	−3

Table S35. Analysis of the free binding energy from the PCI_Lip WT and all exchanges at position S163 for the docking results of pNPH. The empty unit means that substrates couldn't be docked into the active pocket. Affinity at best position means the docking affinity at the minimum distance.

Mutant	Number of meaningful docking results	Minimum distance [Å]	Affinity at best position [kcal/mol]	Highest affinity [kcal/mol]
S163	3	4.5	−5.8	−5.9
S163G				
S163P	4	3.3	−3.9	−5.2
S163L				
S163A				
S163D				
S163E	2	4.7	−6	−6.1
S163I				
S163M	9	4	−5.5	−6
S163N				
S163Q	3	4.4	−5.8	−5.8
S163R				
S163V				
S163W	3	3.5	−5.5	−5.9
S163Y	2	5.6	−4.9	−4.9
S163K				
S163H	3	3.5	−4.9	−5.6
S163C	9	4.2	−5.5	−7.1
S163T	1	6.3	−3.6	−3.6
S163F				

Table S36. Analysis of the free binding energy from the PCI_Lip WT and all exchanges at position S163 for the docking results of pNPO. The empty unit means that substrates couldn't be docked into the active pocket. Affinity at best position means the docking affinity at the minimum distance.

Mutant	Number of meaningful docking results	Minimum distance [Å]	Affinity at best position [kcal/mol]	Highest affinity [kcal/mol]
S163	5	4.5	−5.8	−5.9
S163G				
S163P	4	3.2	−4.3	−5.4
S163L				
S163A	1	4.5	−5.2	−5.2
S163D	2	6.3	−5.7	−6.2
S163E	7	4.2	−5.9	−6.5
S163I				
S163M	5	4.6	−5.8	−6.2
S163N				
S163Q	1	5.9	−6.1	−6.1

S163R				
S163V	1	5.6	−1.1	−1.1
S163W	2	3.8	−6.4	−6.4
S163Y	1	4.4	−5.1	−5.1
S163K				
S163H	2	3.6	−5.2	−5.2
S163C	6	3.8	−5.5	−7
S163T				
S163F	2	4.5	−2.9	−2.9

Table S37. Analysis of the free binding energy from the PCI_Lip WT and all exchanges at position S163 for the docking results of pNPP. The empty unit means that substrates couldn't be docked into the active pocket. Affinity at best position means the docking affinity at the minimum distance.

Mutant	Number of meaningful docking results	Minimum distance [Å]	Affinity at best position [kcal/mol]	Highest affinity [kcal/mol]
S163	6	4.3	−4.2	−5.5
S163G				
S163P				
S163L				
S163A				
S163D	3	3.4	−7	−7
S163E	3	4	−7.2	−7.2
S163I	1	4.2	−5	−5
S163M	7	4.4	−6	−6.6
S163N				
S163Q	8	3.7	−6.5	−6.5
S163R				
S163V	3	4.8	1.6	0.7
S163W	2	3.4	−6.5	−6.5
S163Y	3	5	−2.4	−5.3
S163K	1	6.4	−3.7	−3.7
S163H	4	3.4	−2.8	−3.7
S163C	9	3.7	−6.6	−7.1
S163T	3	3.2	−4.4	−4.4
S163F	2	5.2	−2.7	−2.7

Table S38. Analysis of the free binding energy from the PCI_Lip WT and all exchanges at position I245 for the docking results of pNPA. The empty unit means that substrates couldn't be docked into the active pocket. Affinity at best position means the docking affinity at the minimum distance.

Mutant	Number of meaningful docking results	Minimum distance [Å]	Affinity at best position [kcal/mol]	Highest affinity [kcal/mol]
I245	2	5.1	−4.4	−4.4
I245G				
I245P				
I245L	7	3.9	−5.8	−5.8
I245A				
I245D	6	3.8	−4.3	−6
I245E				
I245M	1	6.3	−5.3	−5.3
I245N				
I245Q	6	3.4	−5.3	−5.6
I245R	1	3.6	−5.4	−5.4
I245S				
I245V				
I245W				
I245Y				
I245K	1	5.7	−5.2	−5.2
I245H	3	3.8	−5.6	−5.6
I245C				
I245T	1	6.5	−4.9	−4.9
I245F	3	5.2	−5.5	−5.5

Table S39. Analysis of the free binding energy from the PCI_Lip WT and all exchanges at position I245 for the docking results of pNPB. The empty unit means that substrates couldn't be docked into the active pocket. Affinity at best position means the docking affinity at the minimum distance.

Mutant	Number of meaningful docking results	Minimum distance [Å]	Affinity at best position [kcal/mol]	Highest affinity [kcal/mol]
I245	3	4.9	−4.8	−5.1
I245G				
I245P				
I245L	11	3.8	−5.7	−6.1
I245A	1	5.8	−5.5	−6.1
I245D	5	5	−5.8	−6.3
I245E				
I245M	2	4.6	−5.5	−5.8
I245N				
I245Q	8	3.4	−5.6	−5.9
I245R	4	3	−5.3	−6.1

I245S				
I245V				
I245W				
I245Y				
I245K	5	3.2	−5.3	−5.6
I245H	5	4	−5.8	−5.8
I245C				
I245T	4	4.1	−5.5	−5.7
I245F	6	3.9	−5.5	−6

Table S40. Analysis of the free binding energy from the PCI_Lip WT and all exchanges at position I245 for the docking results of pNPV. The empty unit means that substrates couldn't be docked into the active pocket. Affinity at best position means the docking affinity at the minimum distance.

Mutant	Number of meaningful docking results	Minimum distance [Å]	Affinity at best position [kcal/mol]	Highest affinity [kcal/mol]
I245	5	4.6	−5.1	−5.1
I245G				
I245P				
I245L	15	3.7	−5.6	−5.8
I245A	2	5.8	−5.4	−5.6
I245D	6	4.7	−6	−6.2
I245E				
I245M				
I245N				
I245Q	12	3.3	−6	−6
I245R	3	3.5	−6.2	−6.5
I245S				
I245V				
I245W				
I245Y				
I245K	2	4.6	−6.2	−6.2
I245H	3	5.1	−5.9	−6.1
I245C				
I245T	3	4.9	−5.3	−5.7
I245F	4	4.4	−5.5	−5.6

Table S41. Analysis of the free binding energy from the PCI_Lip WT and all exchanges at position I245 for the docking results of pNPH. The empty unit means that substrates couldn't be docked into the active pocket. Affinity at best position means the docking affinity at the minimum distance.

Mutant	Number of meaningful docking results	Minimum distance [Å]	Affinity at best position [kcal/mol]	Highest affinity [kcal/mol]
I245	3	4.5	−5.8	−5.9
I245G				
I245P				
I245L	10	3.9	−6.2	−6.4
I245A	1	6	−5.9	−5.9
I245D	4	4.6	−5.4	−6.3
I245E				
I245M				
I245N				
I245Q	12	3.3	−6.3	−6.3
I245R	2	4	−5.9	−5.9
I245S				
I245V				
I245W				
I245Y				
I245K	3	3.4	−5.9	−6
I245H	7	3.9	−5.8	−6.4
I245C				
I245T	3	4.9	−5.6	−6.1
I245F	3	4.9	−6.1	−6.1

Table S42. Analysis of the free binding energy from the PCI_Lip WT and all exchanges at position I245 for the docking results of pNPO. The empty unit means that substrates couldn't be docked into the active pocket. Affinity at best position means the docking affinity at the minimum distance.

Mutant	Number of meaningful docking results	Minimum distance [Å]	Affinity at best position [kcal/mol]	Highest affinity [kcal/mol]
I245	5	4.5	−5.8	−5.9
I245G				
I245P				
I245L	10	4	−6	−6.4
I245A	1	5.9	−5.8	−5.8
I245D	8	4.3	−5	−6
I245E	1	6.4	−2.6	−2.6
I245M	4	4.1	−5.6	−6
I245N				
I245Q	12	3.2	−5.5	−6.4
I245R	4	3	−6.4	−6.9

I245S				
I245V				
I245W				
I245Y				
I245K				
I245H	7	3.9	−6	−6.4
I245C				
I245T	2	5.7	−5.6	−5.6
I245F	4	4.7	−5.8	−5.9

Table S43. Analysis of the free binding energy from the PCI_Lip WT and all exchanges at position I245 for the docking results of pNPP. The empty unit means that substrates couldn't be docked into the active pocket. Affinity at best position means the docking affinity at the minimum distance.

Mutant	Number of meaningful docking results	Minimum distance [Å]	Affinity at best position [kcal/mol]	Highest affinity [kcal/mol]
I245	6	4.3	−4.2	−5.5
I245G				
I245P				
I245L	11	3.8	−6.6	−7.1
I245A	11	4.6	−5.9	−7.1
I245D	3	4.7	−6.9	−6.9
I245E	2	4.7	−1.5	−4.3
I245M	2	4.1	−5.9	−6.1
I245N				
I245Q	10	3.3	−7	−7
I245R	2	3.5	−7.4	−7.4
I245S				
I245V				
I245W				
I245Y				
I245K	1	5.7	−6.4	−6.4
I245H	7	4.8	−6.1	−6.4
I245C				
I245T	3	3.9	−6.3	−6.3
I245F	10	3.5	−6.2	−6.7

Table S44. Analysis of the free binding energy from the PCI_Lip WT and all exchanges at position F129 for the docking results of pNPA. The empty unit means that substrates couldn't be docked into the active pocket. Affinity at best position means the docking affinity at the minimum distance.

Mutant	Number of meaningful docking results	Minimum distance [Å]	Affinity at best position [kcal/mol]	Highest affinity [kcal/mol]
F129	2	5.1	−4.4	−4.4
F129G				
F129P				
F129L	1	3.3	−3.3	−3.3
F129A	1	5.5	−5	−5
F129D				
F129E	2	6.3	−5.5	−5.5
F129I				
F129M	6	3.1	−5.3	−6.1
F129N				
F129Q	6	3.5	−6.3	−6.3
F129R	3	3.3	−5.9	−5.9
F129S				
F129V				
F129W				
F129Y				
F129K	3	3.8	−0.4	−1.8
F129H	2	5.6	−5.3	−5.3
F129C	2	4	−4.7	−4.9
F129T				

Table S45. Analysis of the free binding energy from the PCI_Lip WT and all exchanges at position F129 for the docking results of pNPB. The empty unit means that substrates couldn't be docked into the active pocket. Affinity at best position means the docking affinity at the minimum distance.

Mutant	Number of meaningful docking results	Minimum distance [Å]	Affinity at best position [kcal/mol]	Highest affinity [kcal/mol]
F129	3	4.9	−4.8	−5.1
F129G				
F129P				
F129L	1	3.8	−3.6	−3.6
F129A	4	4.6	−5.1	−5.4
F129D				
F129E	1	6.2	−6.2	−6.2
F129I				
F129M	6	3	−5.6	−6.5
F129N				
F129Q	5	3.5	−6.4	−6.4

F129R	2	4.2	−6.2	−6.2
F129S				
F129V				
F129W				
F129Y				
F129K	3	3.4	−0.7	−0.6
F129H	3	4	−5.7	−5.8
F129C	3	4.5	−4.9	−5.6
F129T				

Table S46. Analysis of the free binding energy from the PCI_Lip WT and all exchanges at position F129 for the docking results of pNPV. The empty unit means that substrates couldn't be docked into the active pocket. Affinity at best position means the docking affinity at the minimum distance.

Mutant	Number of meaningful docking results	Minimum distance [Å]	Affinity at best position [kcal/mol]	Highest affinity [kcal/mol]
F129	5	4.6	−5.1	−5.1
F129G				
F129P				
F129L				
F129A	11	3.9	−5.6	−5.9
F129D				
F129E	3	6.1	−5.9	−5.9
F129I				
F129M	6	3.4	−3.7	−6.8
F129N				
F129Q	6	3.5	−6.7	−6.7
F129R	5	3.3	−6.1	−6.2
F129S				
F129V				
F129W				
F129Y				
F129K	4	3	−0.2	−0.6
F129H	1	4	−5.8	−5.8
F129C	3	3.9	−5.9	−5.9
F129T				

Table S47. Analysis of the free binding energy from the PCI_Lip WT and all exchanges at position F129 for the docking results of pNPH. The empty unit means that substrates couldn't be docked into the active pocket. Affinity at best position means the docking affinity at the minimum distance.

Mutant	Number of meaningful docking results	Minimum distance [Å]	Affinity at best position [kcal/mol]	Highest affinity [kcal/mol]
F129	3	4.5	−5.8	−5.9
F129G				
F129P				
F129L				
F129A	8	3.8	−5.7	−5.8
F129D				
F129E	1	6	−5.5	−5.5
F129I				
F129M	7	3.5	−3.9	−6.9
F129N				
F129Q	7	3.4	−6.9	−6.9
F129R	4	3.2	−6.5	−6.7
F129S				
F129V				
F129W				
F129Y				
F129K	4	3	−0.2	−0.2
F129H	3	4	−6	−6.1
F129C	4	3.8	−6	−6
F129T				

Table S48. Analysis of the free binding energy from the PCI_Lip WT and all exchanges at position F129 for the docking results of pNPO. The empty unit means that substrates couldn't be docked into the active pocket. Affinity at best position means the docking affinity at the minimum distance.

Mutant	Number of meaningful docking results	Minimum distance [Å]	Affinity at best position [kcal/mol]	Highest affinity [kcal/mol]
F129	5	4.5	−5.8	−5.9
F129G				
F129P				
F129L				
F129A	6	3.7	−6.4	−6.4
F129D				
F129E	3	3.5	−3.1	−5.2
F129I				
F129M	5	4.4	−7	−7.1
F129N				
F129Q	8	3.5	−7.3	−7.3

F129R	7	3.5	−7.1	−7.1
F129S				
F129V				
F129W				
F129Y				
F129K				
F129H	4	5	−6.4	−6.9
F129C	7	3	−3.9	−6.3
F129T				

Table S49. Analysis of the free binding energy from the PCI_Lip WT and all exchanges at position F129 for the docking results of pNPP. The empty unit means that substrates couldn't be docked into the active pocket. Affinity at best position means the docking affinity at the minimum distance.

Mutant	Number of meaningful docking results	Minimum distance [Å]	Affinity at best position [kcal/mol]	Highest affinity [kcal/mol]
F129	6	4.3	−4.2	−5.5
F129G				
F129P				
F129L	1	3.1	−3.4	−3.4
F129A	9	4	−6.9	−7
F129D				
F129E	1	5.6	−5.1	−5.1
F129I				
F129M	2	3	−3.1	−3.1
F129N				
F129Q	5	4	−5.6	−7.4
F129R	6	3.2	−7.1	−7.1
F129S				
F129V				
F129W				
F129Y				
F129K				
F129H	12	3.3	−7.4	−7.8
F129C	10	3	−4.4	−6.7
F129T				

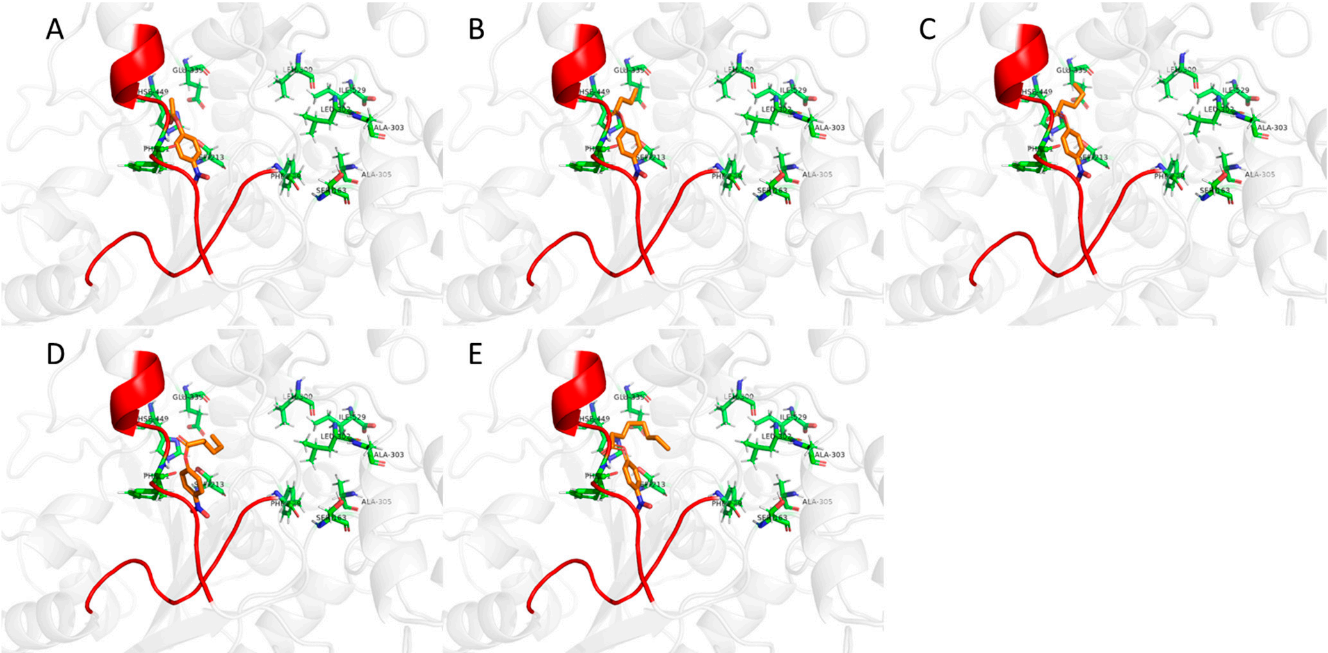

Figure S6. Docking of different chain length *p*-nitrophenol (pNP) esters inside the L305A mutant of the PCI_Lip. Only the best docking results are shown. The criteria of a meaningful docking result is the distance of the S213 towards the carboxyl carbon atom, which were set to be <5.1 Å for pNPA, 4.9 Å for pNPB, 4.6 Å for pNPV, 4.5 Å for pNPH, 4.5 Å for pNPO Å and 4.3 Å for pNPP. The carbon atoms of the protein are colored in green, the nitrogen atoms are blue and the oxygen atoms are colored in red. The carbon atoms of the *p*-nitrophenol (pNP) esters are colored in orange. (**A**) pNP-acetate (pNPA), (**B**) pNP-butyrate (pNPB), (**C**) pNP-valerate (pNPV), (**D**) pNP-hexanoate (pNPH) and (**E**) pNP-octanoate (pNPO). HSE = prototropic tautomer of histidine, H on N2 in the histidine ring, according to the CHARMM force field.

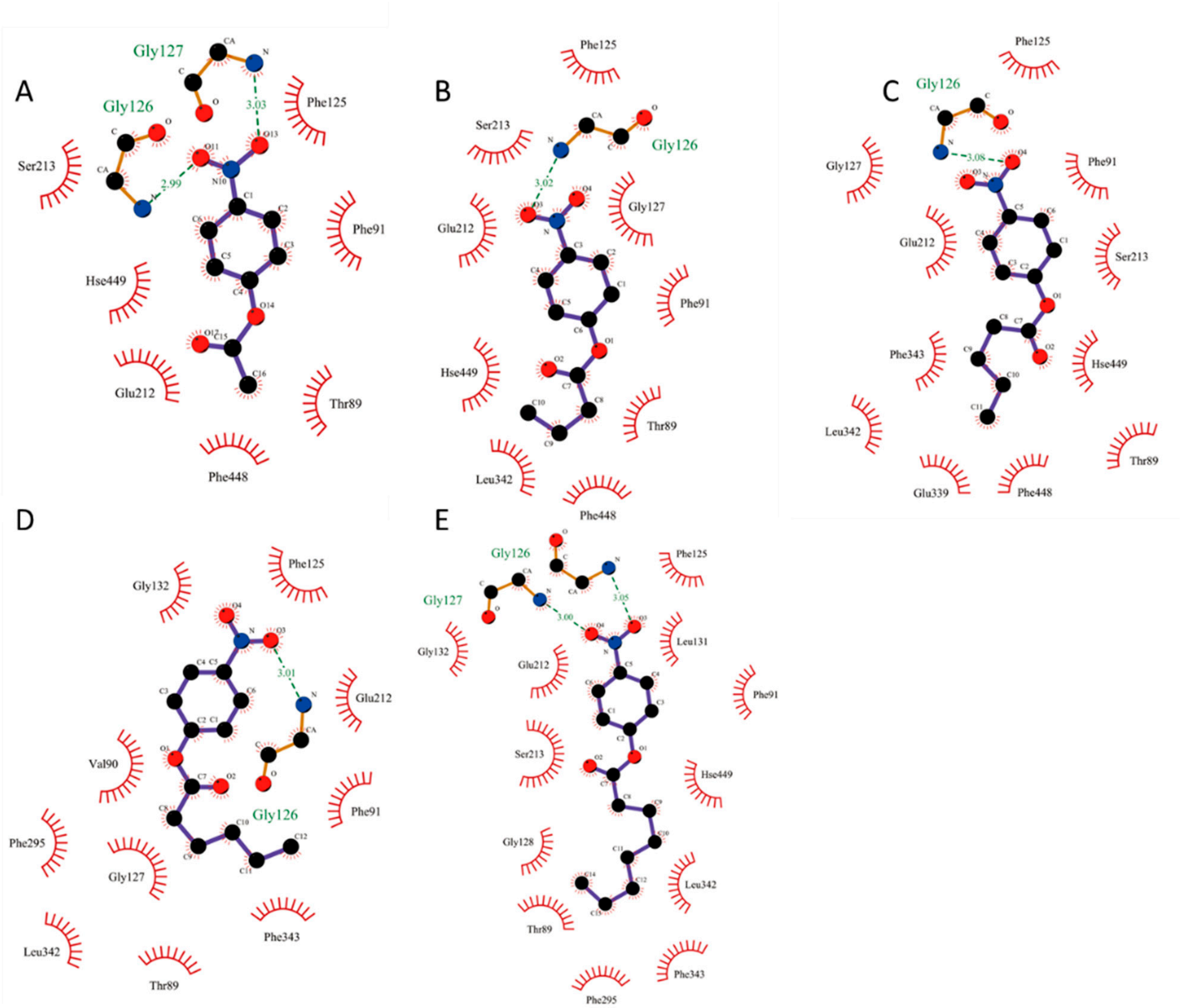

Figure S7. 2D pattern analysis of the docking of different chain length *p*-nitrophenol (pNP) esters inside the L305A mutant of the PCI_Lip generated by using the software LigPloT. Only the best docking results are shown. The residues of the protein which are in the interacting distance are marked with red half circles. The carbon atoms of the *p*-nitrophenol (pNP) esters are colored in black and the bonds in magenta, the nitrogen atoms are blue and the oxygen atoms are colored in red. (**A**) pNP-acetate (pNPA), (**B**) pNP-butyrate (pNPB), (**C**) pNP-valerate (pNPV), (**D**) pNP-hexanoate (pNPH) and (**E**) pNP-octanoate (pNPO). HSE = prototropic tautomer of histidine, H on N2 in the histidine ring, according to the CHARMM force field.

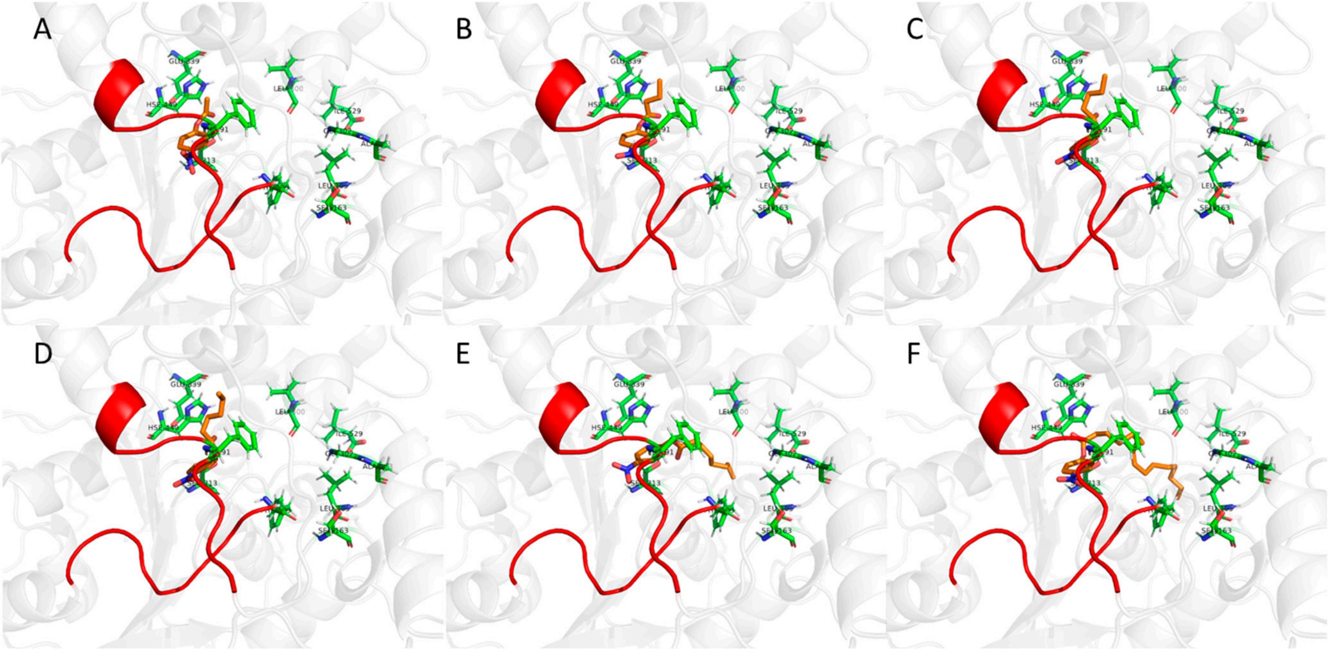

Figure S8. Docking of different chain length *p*-nitrophenol (pNP) esters inside the L302G mutant of the PCI_Lip. Only the best docking results are shown. The criteria of a meaningful docking result is the distance of the S213 towards the carboxyl carbon atom, which were set to be <5.1 Å for pNPA, 4.9 Å for pNPB, 4.6 Å for pNPV, 4.5 Å for pNPH, 4.5 Å for pNPO Å and 4.3 Å for pNPP. The carbon atoms of the protein are colored in green, the nitrogen atoms are blue and the oxygen atoms are colored in red. The carbon atoms of the *p*-nitrophenol (pNP) esters are colored in orange. (**A**) pNP-acetate (pNPA), (**B**) pNP-butyrate (pNPB), (**C**) pNP-valerate (pNPV), (**D**) pNP-hexanoate (pNPH), (**E**) pNP-octanoate (pNPO), and (**F**) pNP-palmitate (pNPP). HSE = prototropic tautomer of histidine, H on N2 in the histidine ring, according to the CHARMM force field.

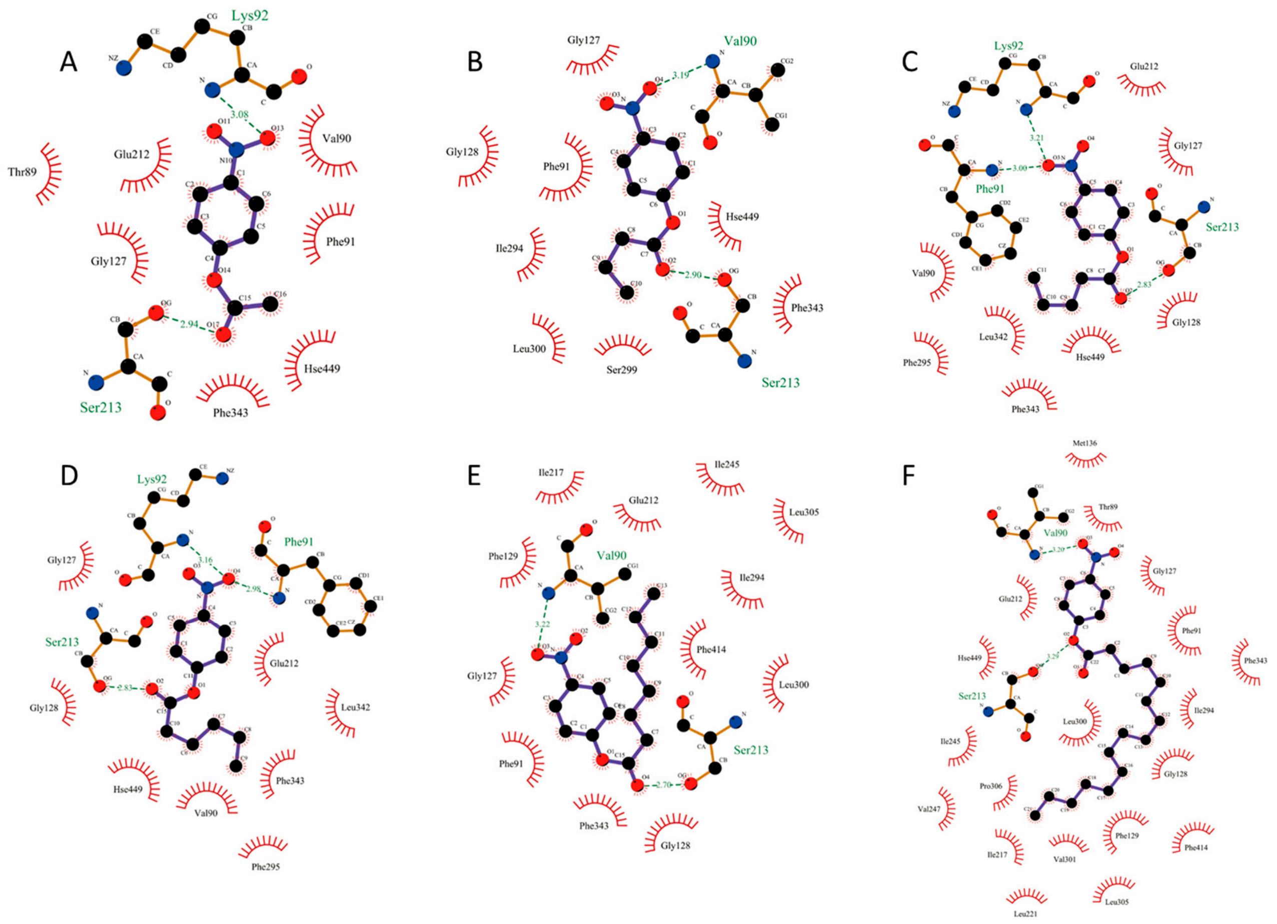

Figure S9. 2D pattern analysis of the docking of different chain length *p*-nitrophenol (pNP) esters inside the L302G mutant of the PCI_Lip generated by using the software LigPloT. Only the best docking results are shown. The residues of the protein which are in the interacting distance are marked with red half circles. The carbon atoms of the *p*-nitrophenol (pNP) esters are colored in black and the bonds in magenta, the nitrogen atoms are blue and the oxygen atoms are colored in red. (**A**) pNP-acetate (pNPA), (**B**) pNP-butyrate (pNPB), (**C**) pNP-valerate (pNPV), (**D**) pNP-hexanoate (pNPH), (**E**) pNP-octanoate (pNPO), and (**F**) pNP-palmitate (pNPP). HSE = prototropic tautomer of histidine, H on N2 in the histidine ring, according to the CHARMM force field.

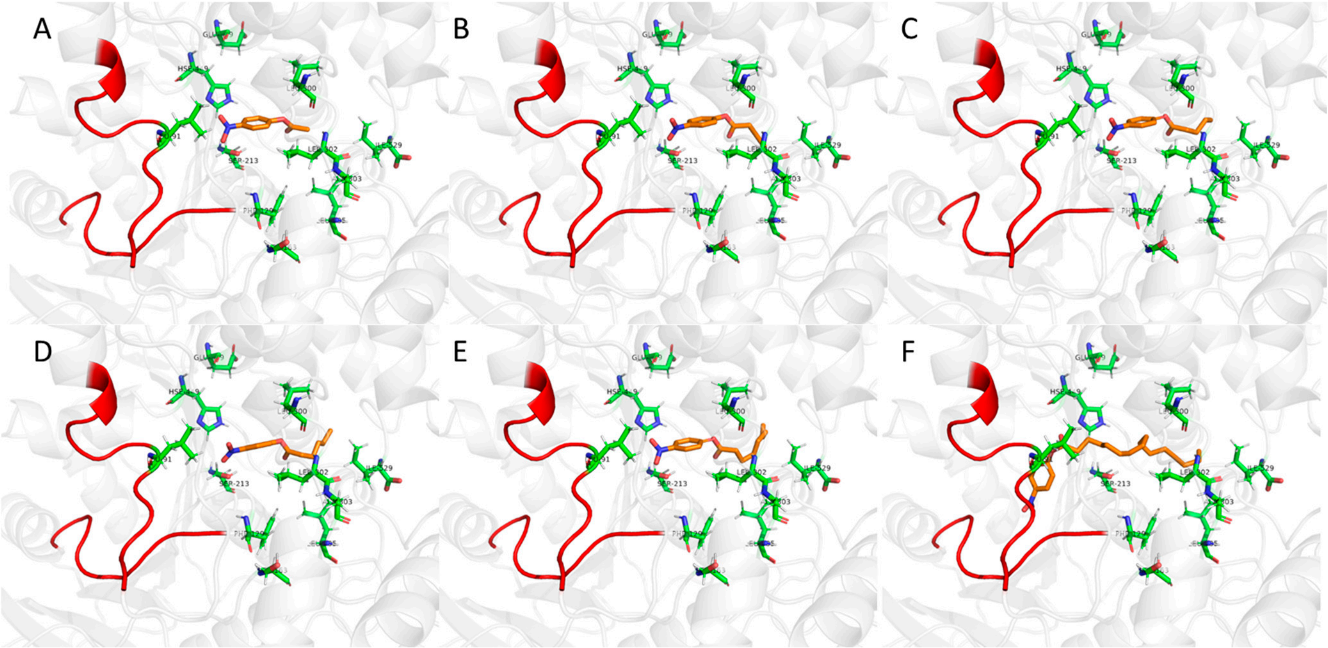

Figure S10. Docking of different chain length *p*-nitrophenol (pNP) esters inside the F91L mutant of the PCI_Lip. Only the best docking results are shown. The criteria of a meaningful docking result is the distance of the S213 towards the carboxyl carbon atom, which were set to be <5.1 Å for pNPA, 4.9 Å for pNPB, 4.6 Å for pNPV, 4.5 Å for pNPH, 4.5 Å for pNPO Å and 4.3 Å for pNPP. The carbon atoms of the protein are colored in green, the nitrogen atoms are blue and the oxygen atoms are colored in red. The carbon atoms of the *p*-nitrophenol (pNP) esters are colored in orange. (**A**) pNP-acetate (pNPA), (**B**) pNP-butyrate (pNPB), (**C**) pNP-valerate (pNPV), (**D**) pNP-hexanoate (pNPH), (**E**) pNP-octanoate (pNPO), and (**F**) pNP-palmitate (pNPP). HSE = prototropic tautomer of histidine, H on N2 in the histidine ring, according to the CHARMM force field.

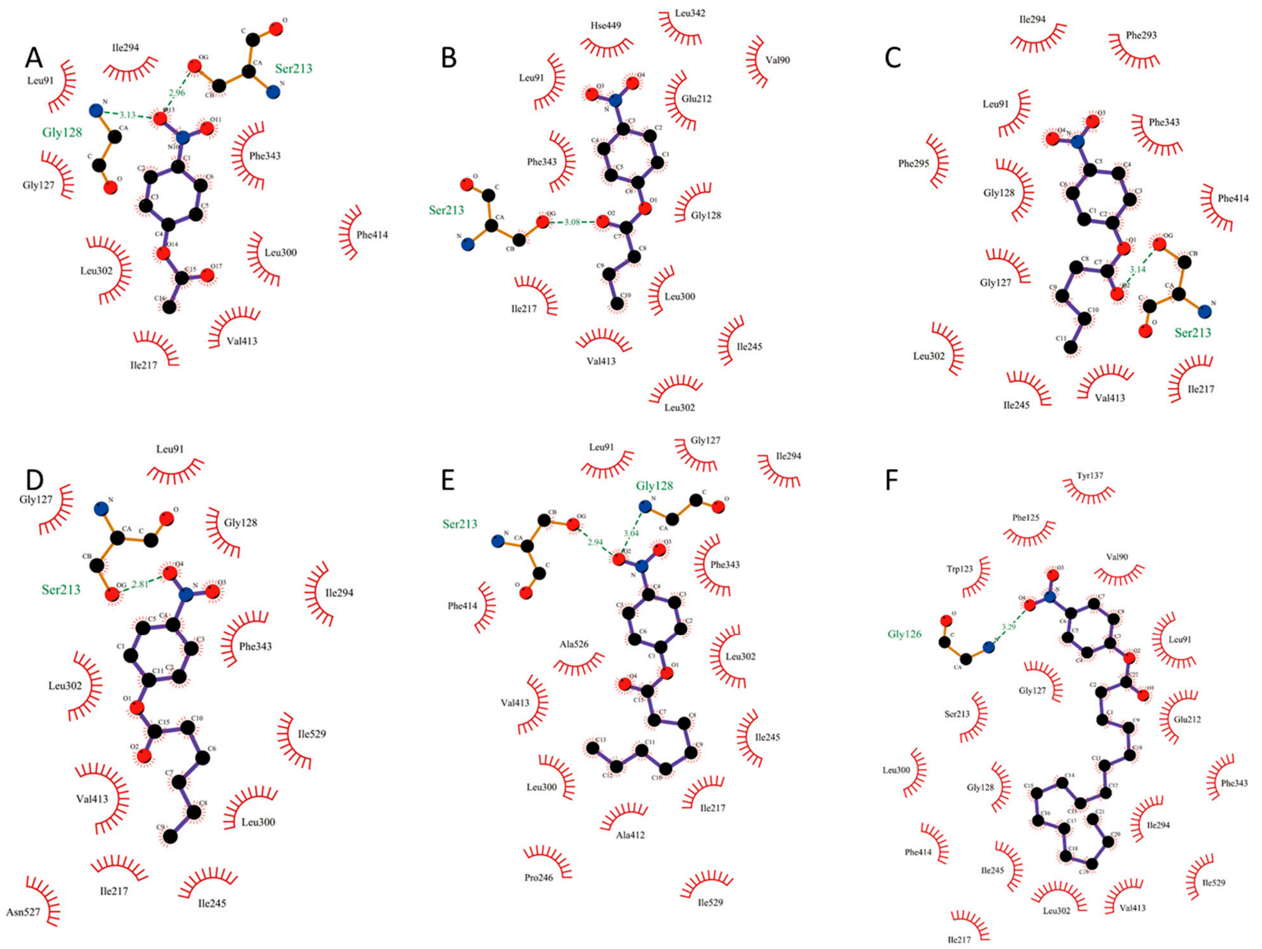

Figure S11. 2D pattern analysis of the docking of different chain length *p*-nitrophenol (pNP) esters inside the F91L mutant of the PCI_Lip generated by using the software LigPloT. Only the best docking results are shown. The residues of the protein which are in the interacting distance are marked with red half circles. The carbon atoms of the *p*-nitrophenol (pNP) esters are colored in black and the bonds in magenta, the nitrogen atoms are blue and the oxygen atoms are colored in red. (**A**) pNP-acetate (pNPA), (**B**) pNP-butyrate (pNPB), (**C**) pNP-valerate (pNPV), (**D**) pNP-hexanoate (pNPH), (**E**) pNP-octanoate (pNPO), and (**F**) pNP-palmitate (pNPP).

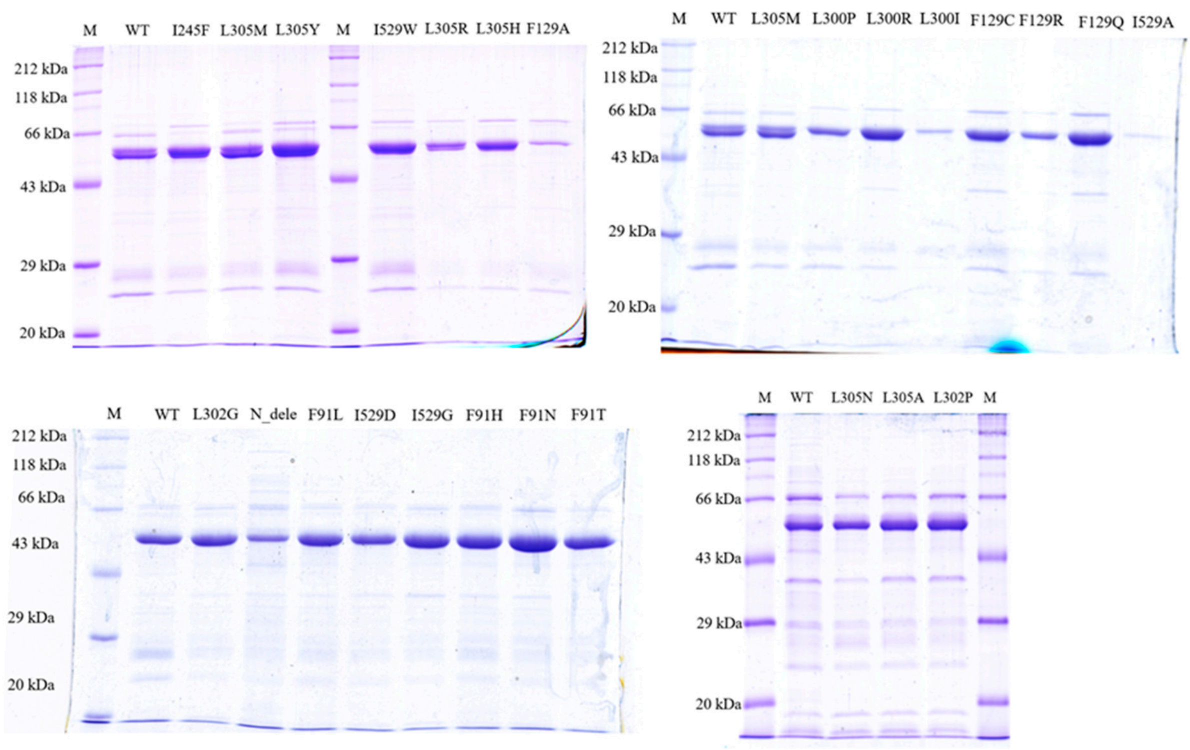

Figure S12. Denaturing SDS-PAGE gels of the IMAC fractions of all expressed PCI_Lip mutants.

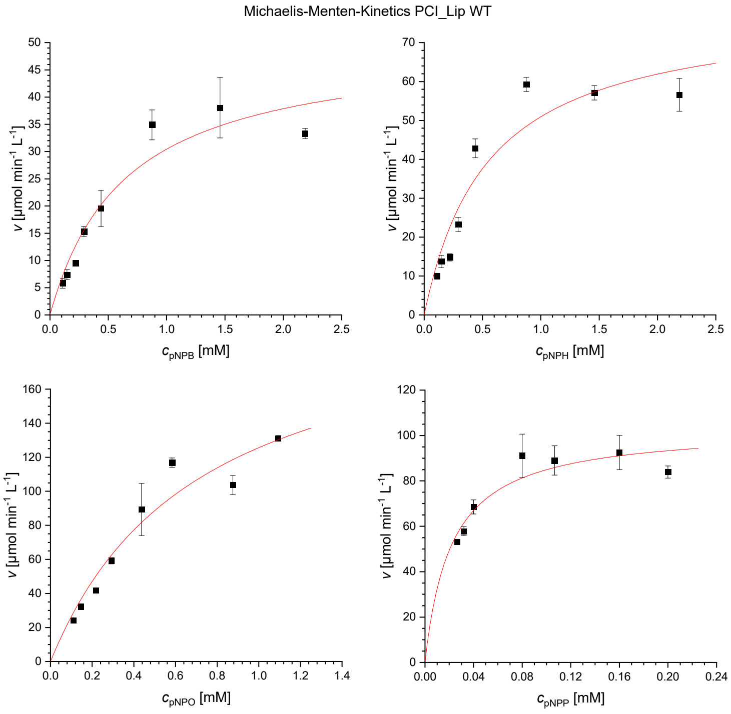

Figure S13. Saturation curves of PCI_Lip WT against selected pNP-Esters (pNPB, pNPH, pNPO and pNPP). Obtained data for the activity were fitted via OriginPro® 2021 based on the Michaelis-Menten equation. In case of pNPB, pNPH and pNPO saturation was not achieved due to a limited solubility of these substrates under the assay conditions. Activity at all concentrations were measured in triplicates and the error bars correspond to standard deviation.

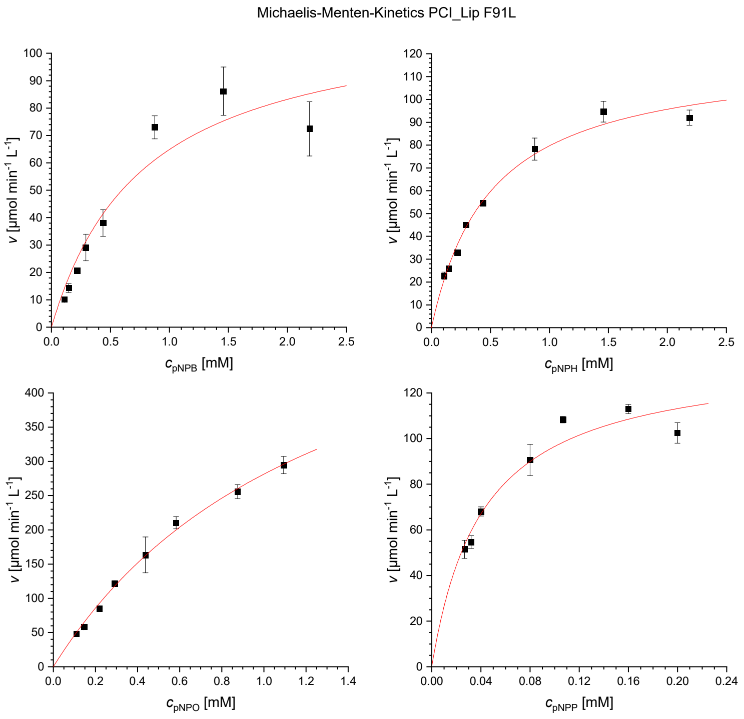

Figure S14. Saturation curves of PCI_Lip mutant F91L against selected pNP-Esters (pNPB, pNPH, pNPO and pNPP). Obtained data for the activity were fitted via OriginPro® 2021 based on the Michaelis-Menten equation. In case of pNPB, pNPH and pNPO saturation was not achieved due to a limited solubility of these substrates under the assay conditions. Activity at all concentrations were measured in triplicates and the error bars correspond to standard deviation.

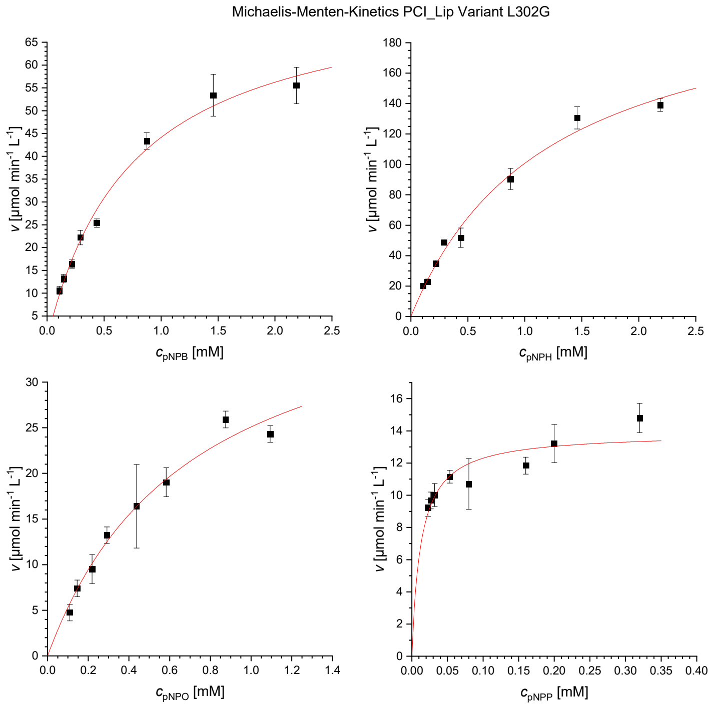

Figure S15. Saturation curves of PCI_Lip mutant L302G against selected pNP-Esters (pNPB, pNPH, pNPO and pNPP). Obtained data for the activity were fitted via OriginPro® 2021 based on the

Michaelis-Menten equation. In case of pNPB, pNPH and pNPO saturation was not achieved due to a limited solubility of these substrates under the assay conditions. Activity at all concentrations were measured in triplicates and the error bars correspond to standard deviation.

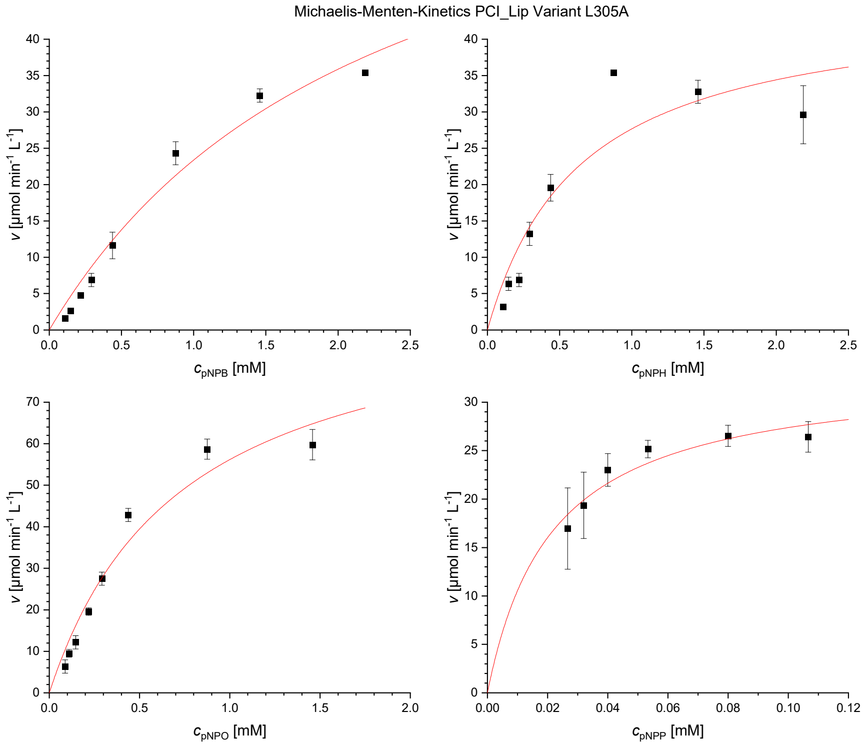

Figure S16. Saturation curves of PCI_Lip mutant L305A against selected pNP-Esters (pNPB, pNPH, pNPO and pNPP). Obtained data for the activity were fitted via OriginPro® 2021 based on the Michaelis-Menten equation. In case of pNPB, pNPH and pNPO saturation was not achieved due to a limited solubility of these substrates under the assay conditions. Activity at all concentrations were measured in triplicates and the error bars correspond to standard deviation.

Table 50. Calculated T_{50}^{60} values and errors for the PCI_Lip WT, F91L, L302G and L305A.

	T_{50}^{60} [°C]
WT	31.5 ± 0.7
F91L	29.8 ± 3.2
L302G	32.9 ± 1.1
L305A	33.5 ± 0.5

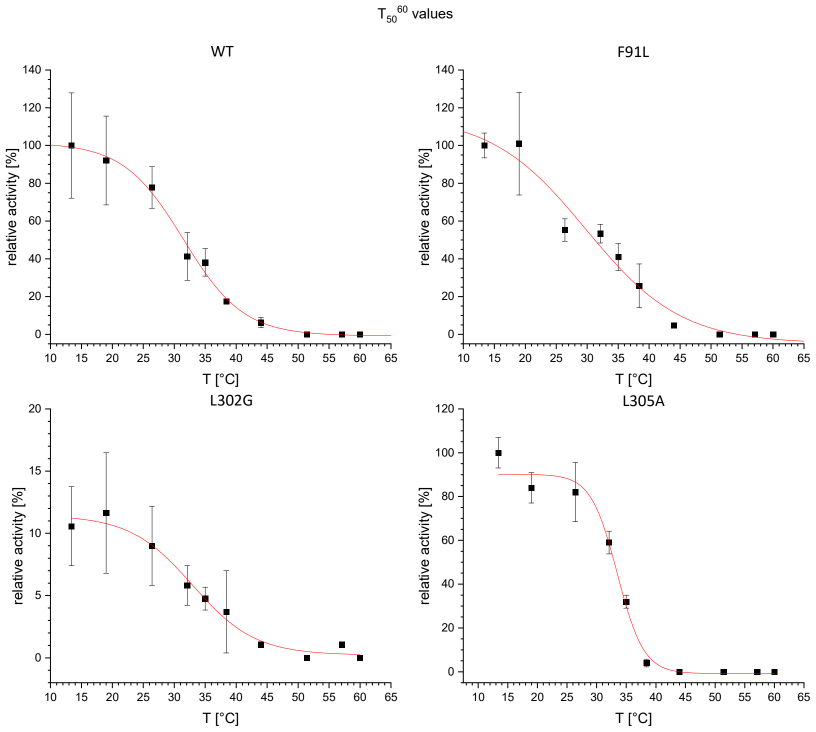

Figure S17. Activities of PCI_Lip WT and mutants F91L, L302G and L305A after incubation for 1 h at different temperatures against pNPO. The activity data was fitted via OriginPro® 2021 using a Boltzman-fit. The turning point of the fitted curve represents the T_{50}^{60} value of the respective PCI_Lip mutant. The T_{50}^{60} value represents the temperature where after a treatment of 1 h 50% of the enzymes activity is lost.

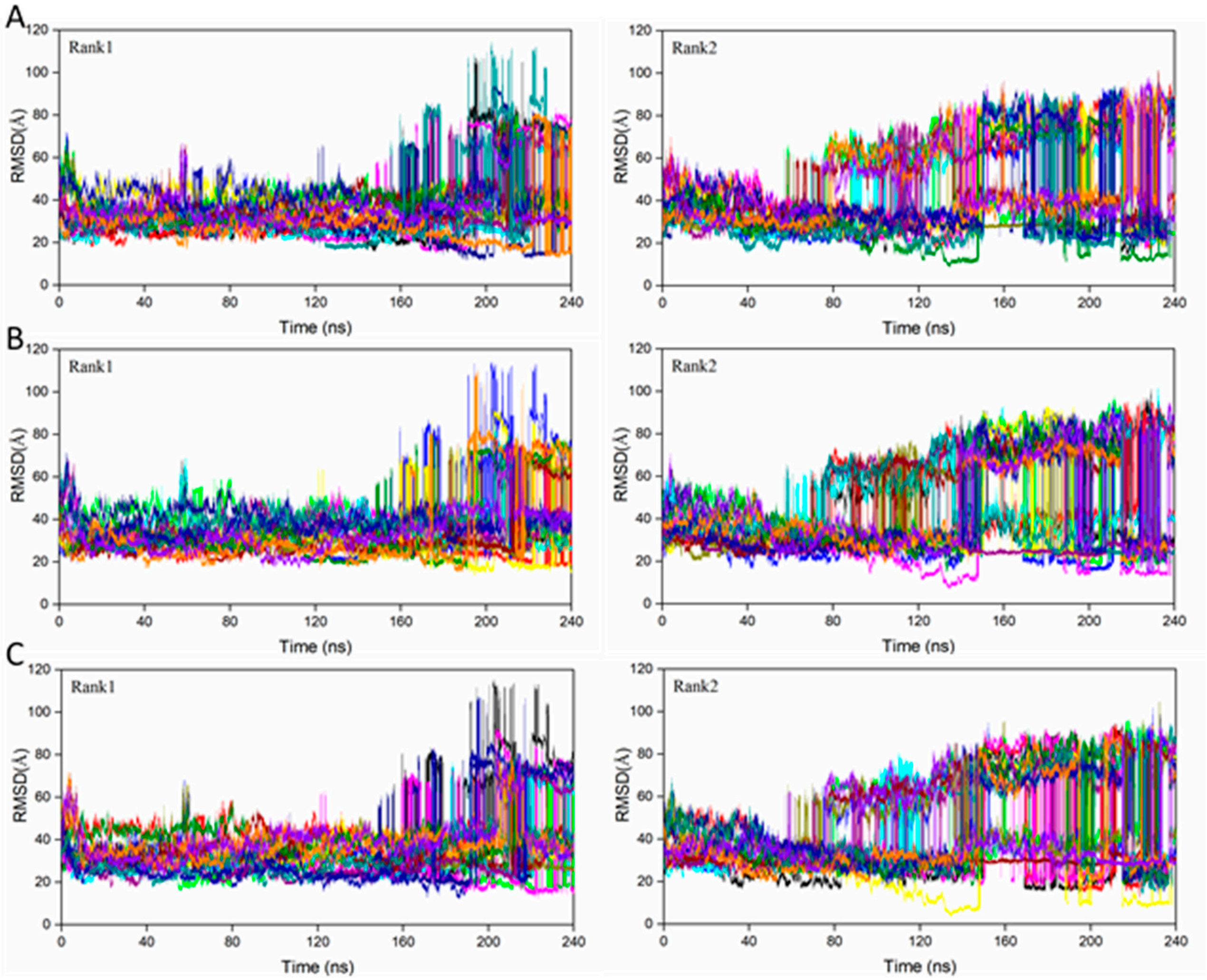

Figure S18. Time resolution of RMSD for wild type PCI_Lip. (**A**) C10:0; (**B**) C6:0; (**C**) C16:0.

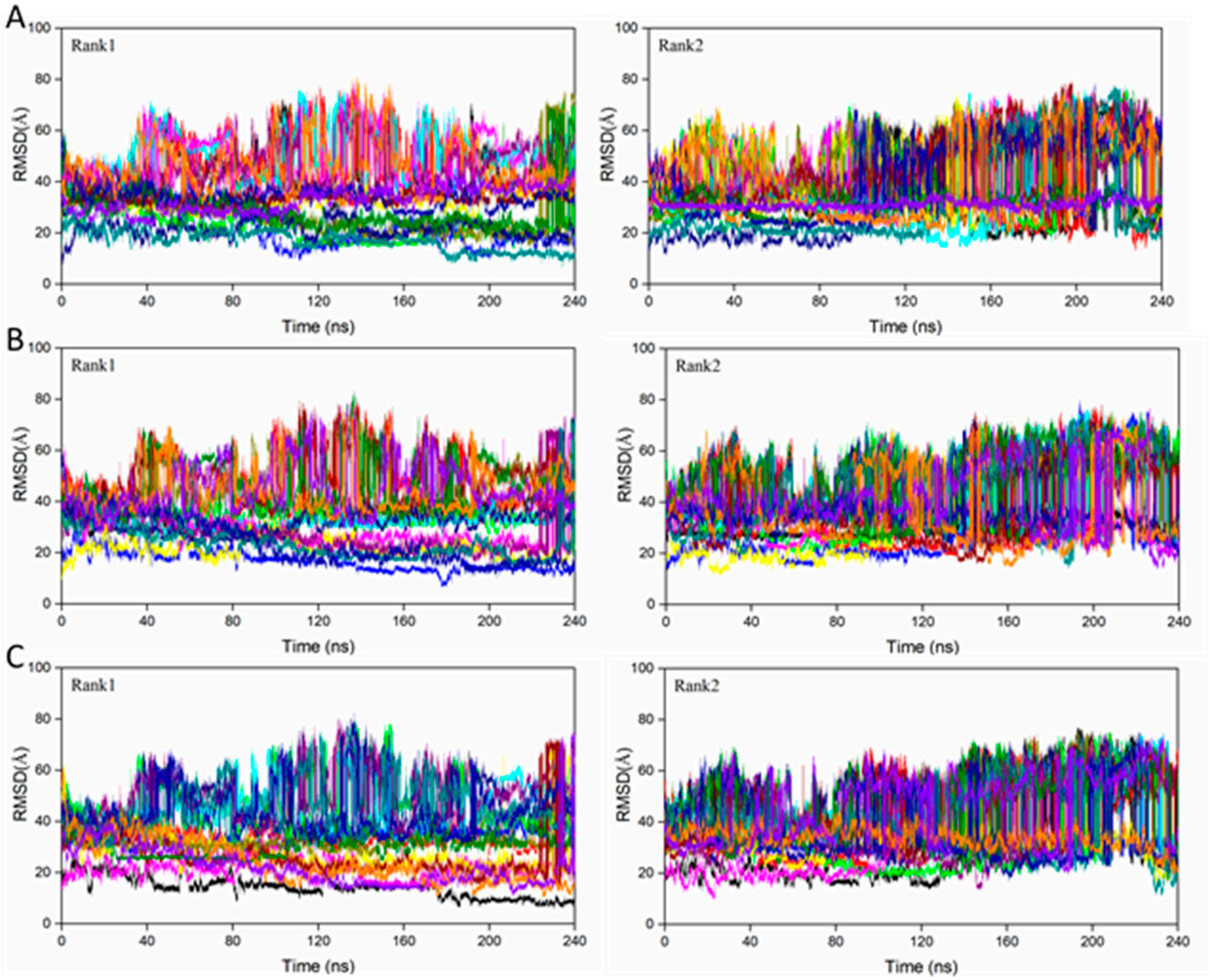

Figure S19. Time resolution of RMSD for L305A mutant of PCI_Lip. (**A**) C10:0; (**B**) C6:0; (**C**) C16:0.

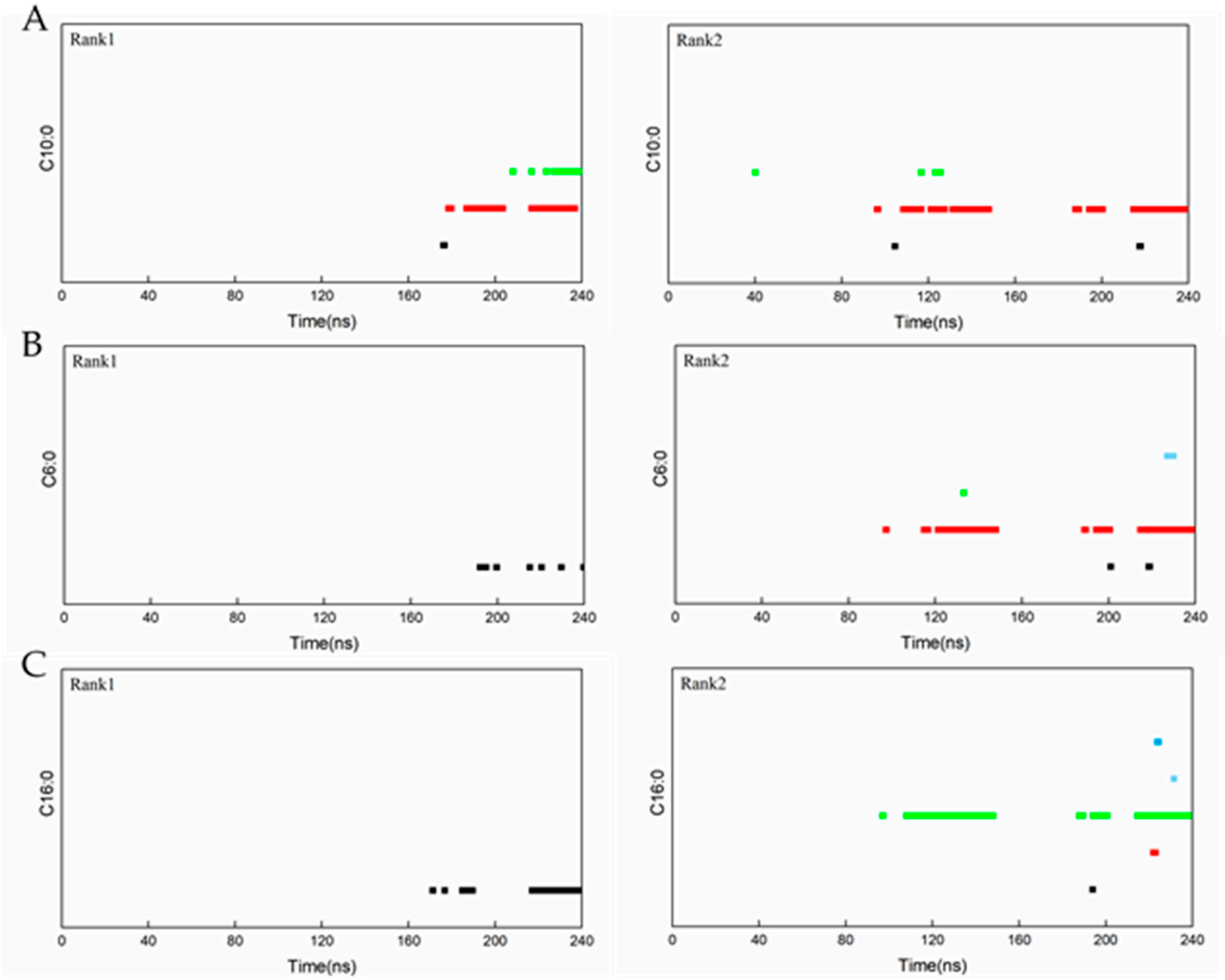

Figure S20. The binding time distribution of fatty acids which could enter the active pocket for wild type PCI_Lip. A kind of color represented one binding time distribution of one fatty acid chain entering the active pocket. (**A**) C10:0; (**B**) C6:0; (**C**) C16:0.

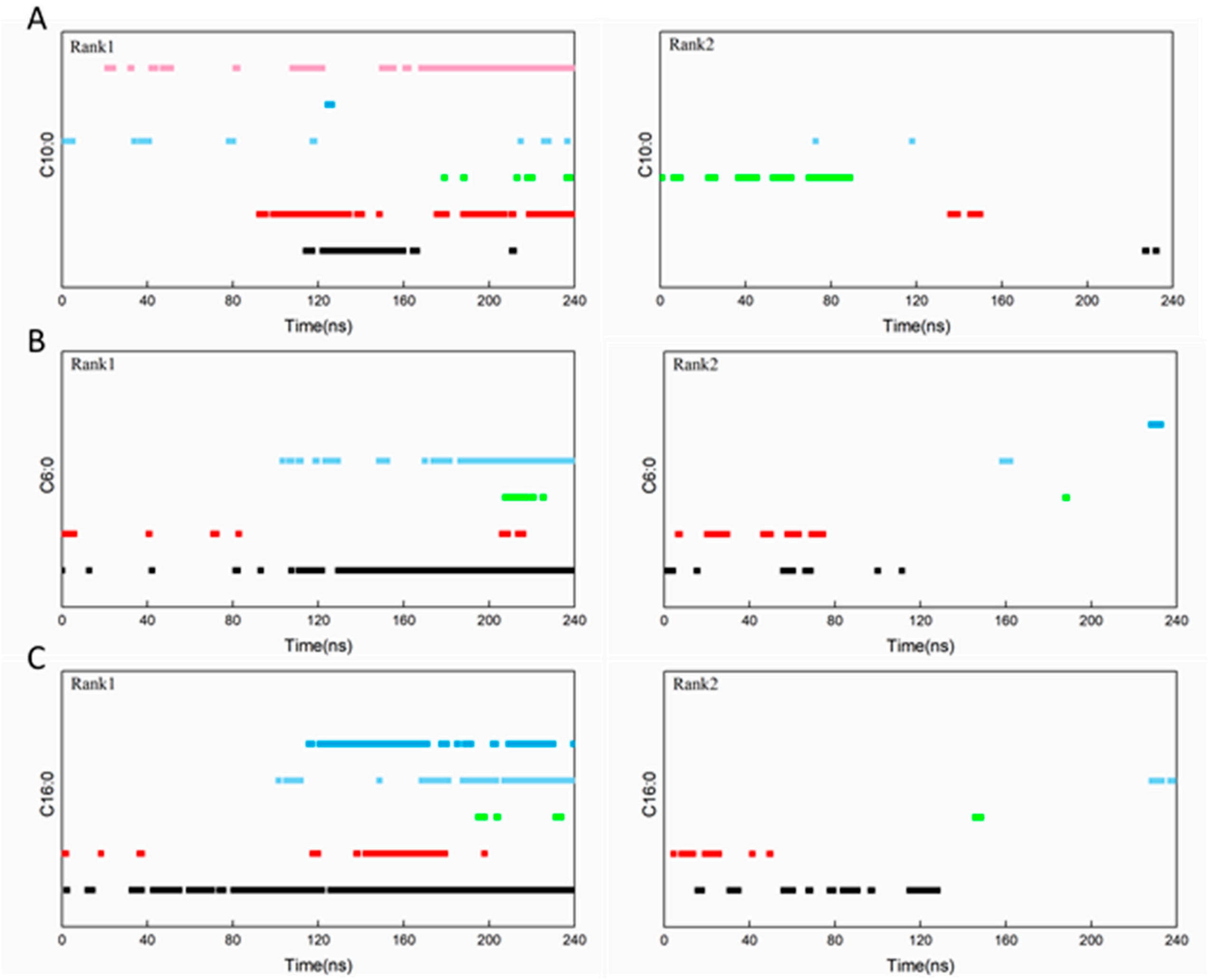

Figure S21. The binding time distribution of fatty acids which could enter the active pocket for L305A mutant of PCI_Lip. A kind of color represented one binding time distribution of one fatty acid chain entering the active pocket. (**A**) C10:0; (**B**) C6:0; (**C**) C16:0.

Table S51. The number and binding time of the fatty acid chains entering the active center of wild type PCI_Lip.

	Fatty acid chain	No.1	No.2
Number	C10:0	3	3
	C6:0	1	4
	C16:0	3	5
Total binding time	C10:0	24.36	43.86
	C6:0	0.42	41.45
	C16:0	4.47	60.12

Table S52. The number and binding time of the fatty acid chains entering the active center of L305A.

	Fatty acid chain	No.1	No.2
Number	C10:0	6	4
	C6:0	4	5
	C16:0	5	4
Total binding time	C10:0	117.6	8.51
	C6:0	130.05	10.86
	C16:0	209.1	11.98
